# ECG-Only Cognitive Workload State Classification in Laparoscopic Training Using Raw and Recurrence-Plot Representations

**DOI:** 10.3390/s26144427

**Published:** 2026-07-12

**Authors:** Kaizhe Jin, Adrian Rubio-Solis, Ravi Naik, George Mylonas

**Affiliations:** 1 Hamlyn Centre for Robotic Surgery, Institute of Global Health Innovation, Imperial College London, London SW7 2AZ, UK; k.jin20@imperial.ac.uk (K.J.); a.rubio-solis@imperial.ac.uk (A.R.-S.); ravi.naik15@imperial.ac.uk (R.N.); 2 Department of Surgery & Cancer, Imperial College London, London SW7 2AZ, UK

**Keywords:** cognitive workload, ECG-only sensing, laparoscopic training, recurrence plots, MultiRocket, transfer learning, SURG-TLX, leave-one-round-out evaluation

## Abstract

Electrocardiography (ECG)-only workload-state classification offers a lower-burden physiological sensing route than denser multimodal, multi-sensor physiological, or neuroimaging setups for controlled laparoscopic training research. This study evaluated whether ECG-only representations can classify condition-derived cognitive workload states during a controlled laparoscopic peg transfer task performed under Control and auditory N-back conditions (N0, N1, and N2). Twenty surgical trainees from an advanced surgical-skills course completed the task protocol, and 17 participants entered ECG modelling after ECG quality-control exclusions. The retained ECG modelling dataset comprised 268 task blocks (Control/N0/N1/N2: 68/68/68/64), evaluated as held-out task-block predictions in a known-participant four-fold leave-one-round-out (LOTO) evaluation. Branch-specific raw ECG windows, recurrence-plot sequences, and heart-rate/time-domain heart-rate-variability inputs are detailed in the Methods, and model metrics were computed after reduction to task-block predictions. The primary endpoint was Surgery Task Load Index (SURG-TLX)-aligned low/high workload, defined as Control plus N0 versus N1 plus N2. Four-class and three-level endpoints were retained as secondary views. Raw ECG, recurrence-plot (RP)-derived, hybrid score-level fusion, and conventional heart-rate/time-domain heart-rate-variability Random Forest (HRV-RF) models were compared using a locked evaluation protocol, leakage-aware train-fold-only preprocessing, participant-clustered confidence intervals, and planned paired tests for the primary endpoint. On the primary low/high endpoint, raw ECG achieved the highest macro-F1/balanced accuracy (0.865/0.865), followed by the hybrid branch (0.847/0.847) and HRV-RF (0.648/0.649). Raw ECG and hybrid were supported over RP-derived and HRV-RF under the planned paired tests. On selected secondary endpoints, hybrid achieved higher macro-F1 values than raw ECG, consistent with possible endpoint-dependent RP-derived complementarity rather than a general hybrid advantage. These findings support ECG-only block-level workload-state classification in this controlled training setting. The evidence is retrospective and based on held-out rounds from known participants rather than subject-independent or deployment validation.

## 1. Introduction

Surgical performance in operating and training environments requires technical execution under perceptual, motor, and decision-making demands. Cognitive workload (CWL) is therefore important for understanding how surgeons and trainees manage task demands while maintaining safe and effective performance [1]. Controlled laparoscopic training tasks provide a reproducible setting in which these demands can be manipulated, measured, and modelled outside live patient care. In such tasks, workload demands are intensified by indirect visualisation, constrained instrument motion, depth judgement, and the need to respond to stress or distraction [2,3]. Workload assessment can support performance analysis, training-task evaluation, and objective support tools for surgical education [1].

A central challenge in this area is how workload is measured. Surgical and neurophysiological workload reviews describe CWL as a construct inferred from subjective ratings, behavioural performance, and physiological signals, rather than as a quantity captured by one definitive physiological reference signal [4,5,6]. Subjective workload instruments are widely used because they are simple to administer and provide interpretable ratings of perceived task demand. NASA Task Load Index (NASA-TLX) provides a general workload measure, while Surgery Task Load Index (SURG-TLX) was developed to capture demand sources that are more specific to operative tasks [7,8]. These instruments are valuable in surgical workload studies, but they are retrospective and task-level measures. Behavioural summaries and physiological signals can therefore complement subjective ratings by showing how workload-related demand appears during task execution.

This measurement challenge explains why physiological sensing has become increasingly important in CWL studies. Physiological signals can provide objective, time-resolved measurements during task performance, whereas subjective scales are collected after a task period. They do not replace perceived workload ratings, but they can provide a contemporaneous signal route for modelling workload-related changes during structured training. This is particularly relevant in surgical training, where workload may fluctuate within a task while visual attention and manual control must be maintained.

The way workload is induced is closely related to this measurement problem. If task-condition labels are used for workload-state classification, the underlying manipulation should create reproducible changes in demand while remaining relevant to surgical training. Surgical tasks often require the operator to maintain a primary visuomotor activity while processing additional information, distractions, time pressure, or communication demands [1,3,9]. In studies of surgical CWL, a controlled laboratory task cannot reproduce the full operating room. It can, however, reproduce a primary-task plus secondary-demand structure in a repeatable way. N-back paradigms are widely used to impose graded demands on working memory and cognitive load. Figalová et al. reported that a higher N-back condition increased perceived workload and altered neurophysiological responses, and Khan et al. used auditory N-back as a secondary cognitive-load task during a primary task [10,11]. Pairing laparoscopic peg transfer with an auditory N-back task therefore provides a controlled multitask workload manipulation for structured surgical training. Peg transfer preserves the primary laparoscopic motor task, while auditory N-back adds graded working-memory demand without drawing visual attention away from the laparoscopic display. SURG-TLX ratings and task-performance summaries can then assess how the manipulation appears in subjective and behavioural evidence, while electrocardiography (ECG) provides the physiological signal route for classification.

The sensing route must also be practical for repeated surgical training. Recent cognitive-load datasets and surgical CWL studies show the value of rich multimodal sensing. CLARE combined ECG, electrodermal activity, electroencephalography (EEG), and gaze for multimodal cognitive-load assessment, and recent laparoscopic CWL work used multimodal physiological and behavioural streams to classify workload during surgical tasks [9,12]. These studies show what can be gained from richer sensing. They also highlight the practical cost of dense instrumentation. Additional sensors can increase preparation time, reduce comfort, restrict movement, and increase signal-loss risk during clinical or training performance [13]. This trade-off is especially important in laparoscopic training, where sensing should not interfere with movement, visual attention, or task execution.

Evidence from multimodal laparoscopic CWL work further motivates the ECG-only question. Naik et al. recorded ECG alongside EEG, functional near-infrared spectroscopy (fNIRS), electromyography (EMG), and pupil diameter in a simulated laparoscopic cholecystectomy study, and their feature-selection analysis retained ECG within a reduced high-performing multimodal subset [9]. Their study, therefore, suggests that ECG can carry workload-relevant information inside a richer sensing pipeline. It does not establish that ECG alone is sufficient, but it makes ECG a plausible candidate for a lower-burden workload-state classifier. ECG-only sensing is attractive because it can be collected with compact hardware and analysed as waveform data or through conventional ECG-derived summaries such as heart rate (HR) and heart-rate variability (HRV) [14,15,16].

The lower-burden ECG-only route then raises a modelling question: how should the ECG signal be represented? A recent systematic review of physiological-signal machine learning reported a shift from shallow machine learning (ML) to deep learning (DL) for cognitive-state recognition, with DL becoming dominant by 2023 and ECG identified as the most frequently used modality [17]. The same review reported that convolutional neural networks (CNNs) were the most common DL approach in real-world cognitive-state studies, appearing in 34 of 55 studies, and that 68% of ECG-utilising studies used CNN-based architectures [17]. This trend is relevant because CNNs are well-suited to spatial feature extraction and are often used when physiological time series are converted into image-like formats. For ECG workload-state classification, this motivates comparison between conventional feature routes, raw waveform modelling, and image-like transformations.

Raw ECG, HR/HRV summaries, and recurrence-plot (RP) representations provide distinct routes through the same ECG signal. HR/HRV summaries provide compact ECG-derived features and support conventional comparator models. Raw ECG retains waveform morphology and temporal structure with limited manual feature design. Recurrence plots re-express the same ECG waveform through temporal recurrence structure, creating an image-like representation without adding a new sensor channel [18,19]. Recent ECG studies have used recurrence-plot images with deep-learning models, supporting RP-derived encoding as a plausible representation route [20]. If raw waveform and RP-derived recurrence structure capture complementary information, score-level fusion provides a direct way to test that complementarity. What remains unclear is whether recurrence information adds value beyond raw ECG for surgical workload-state classification, especially when broad low/high boundaries and finer workload-state distinctions are both considered.

The evaluation setting is also part of the research problem. Surgical training and learning-curve assessment often involve repeated attempts by known trainees or surgeons under changing demands [2,21]. For this use case, generalisation across held-out task rounds from known participants is an important validation question. It tests whether a calibrated model carries across repeated attempts within the same trainee cohort. This differs from subject-independent validation, which asks whether a model transfers to unseen users. Both forms of validation are useful, but they address different stages of translation. A calibrated-user held-out-round design is therefore appropriate for evaluating repeated structured training assessment when it is reported with leakage-aware preprocessing and participant-clustered uncertainty.

Together, this literature defines a specific gap for structured laparoscopic training. Prior surgical CWL studies support physiological workload modelling, and multimodal approaches show the value of rich sensing, but it remains unclear whether a lower-burden ECG-only route retains enough workload-relevant information for this setting. ECG and HRV studies support conventional feature modelling, but they do not resolve how HR/HRV features compare with raw ECG and image-like ECG representations under the same surgical-training endpoints. Representation-learning studies motivate deep and image-like encodings, but they do not show whether recurrence information is useful beyond raw ECG for this task. Repeated-training studies motivate held-out-round evaluation, but this calibrated-user evidence must be distinguished from subject-independent or deployment-focused validation.

This study had three objectives. First, it tested whether ECG-only signals can support condition-derived workload-state classification during controlled laparoscopic peg transfer and auditory N-back training, including performance across held-out task rounds from known participants. Second, it compared raw ECG, RP-derived, hybrid score-level fusion, and a heart-rate/time-domain heart-rate-variability Random Forest (HRV-RF) comparator across a SURG-TLX-aligned primary low/high endpoint and secondary finer endpoints. Third, it evaluated these comparisons using leakage-aware preprocessing, participant-clustered uncertainty, and planned paired comparisons. Together, these objectives examine whether a lower-burden ECG-based route can support structured surgical training assessment and whether RP-derived recurrence information provides useful complementarity beyond raw ECG for finer workload boundaries.

## 2. Materials and Methods

### 2.1. Study Design and Workload Conditions

**Figure 1:**
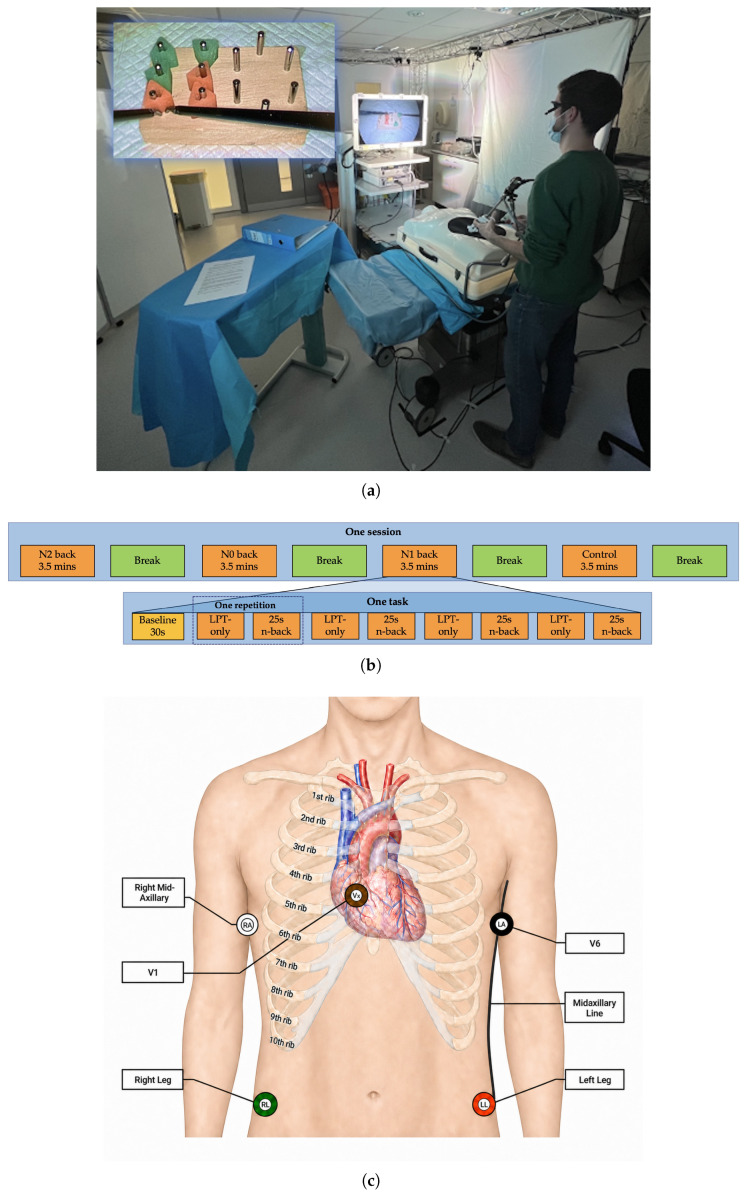
Experimental setup, protocol structure, and ECG electrode placement for the controlled laparoscopic workload study. (**a**) Experimental setup for the laparoscopic peg transfer task. (**b**) Example session structure showing baseline, task cycles, and randomised workload conditions (LPT = laparoscopic peg transfer). (**c**) ECG electrode placement used for acquisition.

The study used a within-subject repeated-measures design to induce graded CWL during a simulated laparoscopic peg transfer task. Workload condition was the experimental independent variable, with four levels: Control, N0, N1, and N2. The dependent measures were post-condition SURG-TLX scores, peg transfer task-performance summaries, N-back accuracy where applicable, and ECG-derived classification outcomes. The peg transfer task was selected because it is a standard component of the Fundamentals of Laparoscopic Surgery skills framework and is widely used in structured laparoscopic skills training [2]. It requires bimanual coordination, depth judgement, and controlled instrument handling under indirect visualisation. A box trainer with a bespoke pegboard, fixed 2D zero-degree 10 mm laparoscope, and VISERA ELITE system (Olympus Medical Systems Corp., Tokyo, Japan) were used, with the monitor at head height to standardise posture (Figure 1a).

During the primary task, participants relocated six pegs between predefined positions on the pegboard. Each transfer required the participant to lift the peg with the grasper on the same side as the starting position, transfer it in mid-air to the opposite grasper, and place it on the target position. Dropped pegs and handling errors were recorded by a clinical research fellow.

Graded workload was induced by combining the peg transfer task with an auditory N-back working-memory task. The peg transfer task was the primary laparoscopic motor task. The auditory N-back task was the secondary working-memory task, selected because N-back paradigms are recognised manipulations of working memory and cognitive-load demand [10,11,22]. This primary-task plus secondary-demand structure provided a repeatable multitask analogue for structured surgical-training CWL research. Control denoted peg transfer only, whereas N0, N1, and N2 paired peg transfer with the auditory task at N = 0, N = 1, and N = 2, respectively. These labels represent designed workload conditions rather than direct measurements of latent CWL.

Each session contained the four workload conditions in randomised order. Each condition began with a 30 s resting baseline, during which participants rested with eyes closed and hands on the laparoscopic graspers. For N-back conditions, the baseline was followed by 3.0 min of task activity organised as four 45 s cycles. Each cycle contained 20 s of peg transfer only, followed by 25 s of concurrent peg transfer and auditory N-back. The 20 s peg transfer-only interval was treated as a lead-in interval and was not retained as an N-back-labelled ECG modelling block. The retained N0, N1, and N2 ECG modelling blocks therefore came from the concurrent 25 s intervals, while Control used matched peg transfer-only intervals. Each participant was scheduled to contribute up to four retained ECG task blocks per condition. Thus, peg transfer-only lead-in intervals were not assigned N0, N1, or N2 labels. Window-level ECG segments were model inputs and were reduced to task-block predictions before performance reporting, as described in Section 2.5 and Section 2.11. Overlapping windows were not treated as independent evaluation units. The N-back stimuli were presented auditorily, and participants responded verbally to reduce gaze shifts away from the laparoscopic monitor. The baseline periods, task cycles, and randomised workload-condition structure are shown in Figure 1b. ECG electrode placement for acquisition is shown in Figure 1c, with acquisition details provided in Section 2.3.

### 2.2. Participants and Ethics

Twenty surgical trainees were recruited during an advanced surgical skills course and contributed subjective workload records. Inclusion required prior laparoscopic experience. Participants with relevant medical conditions, those taking regular medication that could affect physiological measurements, and those with uncorrected vision were excluded to reduce avoidable physiological and performance-related confounding. Table 1 summarises the recruited cohort and the ECG modelling cohort.

The ECG modelling analyses used 17 participants after signal-quality and timestamp-based exclusions. These exclusions followed the predefined ECG quality-control workflow and were applied before classifier training or model comparison. P004 and P019 were excluded because of ECG signal drop-off, and P006 was excluded because of ECG channel-quality issues. P020 was retained for Control, N0, and N1 but contributed no retained N2 ECG modelling blocks because the P020 N2 recording was affected by a documented ShimmerECG timestamp gap. The exclusions were based on signal-quality or timestamp criteria and were independent of classifier outputs, branch performance, and endpoint-specific results. Table 1 reports the recruited and ECG modelling cohorts side by side to show the cohort distribution after ECG quality-control exclusions. The retained ECG modelling data comprised 268 task blocks: 68 Control, 68 N0, 68 N1, and 64 N2. Dense raw ECG window counts are reported with the preprocessing description in Section 2.5.

Ethical approval was granted by the Imperial College Research Governance and Integrity team (ICREC No.: 20IC6361), and all participants provided written informed consent. The study followed institutional and international ethical principles for research involving human participants.

### 2.3. Data Acquisition

Data streams and task events were recorded and synchronised through the Multi-sensing AI Environment for Surgical Task and Role Optimisation (MAESTRO) acquisition framework, which provided time-aligned physiological streams and experiment-control event markers [9,23]. The experiment script generated markers for baseline periods, task onset, and task end. These markers were used consistently across participants and sessions and provided the acquisition-level boundaries for later ECG cropping, segmentation, and alignment with condition-derived workload labels.

Although multiple sensing streams were recorded for experiment monitoring and quality assurance, the classification analyses in this article used ECG only. ECG was acquired using a wireless Shimmer3 ECG Unit (Shimmer Research Ltd., Dublin, Ireland) [24]. The Shimmer3 ECG sampling rate was set to 512 Hz to provide temporal detail for downstream windowing and representation extraction. A five-electrode configuration was used, with electrodes placed on the right arm, left arm, right leg, left leg, and the Vx chest position, as shown in Figure 1c. Skin sites were cleaned with alcohol wipes before electrode placement, and signal quality was checked after acquisition began. Sensors were adjusted when required to improve contact quality and signal stability. The analysed ECG channels were left arm–right arm (LA–RA), left leg–right arm (LL–RA), and Vx–right leg (Vx–RL). These channels were simultaneously available from the five-electrode Shimmer ECG configuration and provided a fixed three-channel input for the raw ECG and RP-derived representations described in Section 2.5. The fixed channel set supported representation comparability and was not selected separately by participant, condition, or held-out model performance. For the HR/HRV comparator, the same channels were treated as candidates for the fold-specific channel-selection procedure described in Section 2.7.

### 2.4. Subjective Workload and Task Performance Measures

Subjective workload was measured using SURG-TLX, a surgery-specific workload questionnaire [8]. Participants completed SURG-TLX after each task condition, so ratings reflected perceived workload for the immediately preceding exposure. The total SURG-TLX score was used as the primary subjective workload measure supporting the endpoint-rationale analysis described in Section 2.8. SURG-TLX was not used as an ECG model input, tuning criterion, or branch-selection metric.

Task performance during peg transfer was quantified as the number of pegs successfully transferred and the number of performance errors. Errors comprised dropped pegs and handling errors, including failure to complete the mid-air transfer step or moving a peg with the wrong grasper. These measures were recorded live and verified by post hoc video analysis. N-back response accuracy was available for N0, N1, and N2. Peg transfer summaries and N-back accuracy are reported by condition in Section 3.1 as behavioural manipulation-check evidence. They provided context for the intended workload and task-difficulty gradient, but they were not used to assign ECG labels, tune models, or select model branches. The endpoint hierarchy was defined from the experimental workload-condition structure. SURG-TLX evidence was used to assess alignment with perceived workload, not to assign ECG labels or tune model branches, as described in Section 2.8.

For subjective workload analysis, total SURG-TLX scores were compared across Control, N0, N1, and N2 using a repeated-measures analysis in the ECG modelling cohort. The planned low/high contrast compared each participant’s mean score for Control plus N0 with the corresponding mean for N1 plus N2 using a paired *t*-test, with Cohen’s $d_{z}$ reported where applicable. Exploratory or supporting adjacent and endpoint-aligned contrasts were interpreted separately from the planned low/high contrast, with Holm adjustment applied where those contrast families were reported. N-back accuracy was analysed for N0, N1, and N2 as a task-performance manipulation check. Peg transfer and failed-transfer summaries were reported descriptively to characterise task performance. Assumption checks were performed for the repeated-measures analyses: sphericity was assessed using Mauchly’s test, and the Greenhouse–Geisser correction was used when sphericity was not supported. The planned low/high SURG-TLX contrast was checked using Shapiro–Wilk testing of paired differences and Wilcoxon signed-rank sensitivity analysis, while the bounded N-back manipulation-check analysis was interpreted using Greenhouse–Geisser correction and Friedman sensitivity analysis where appropriate.

### 2.5. ECG Preprocessing and Representation Branches

**Figure 2:**
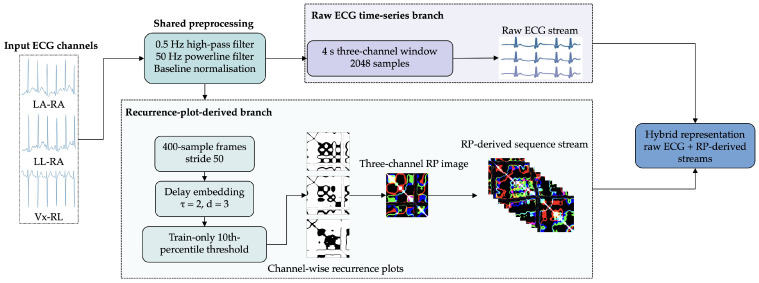
ECG preprocessing and representation branches. Raw ECG samples were converted to calibrated millivolt traces before cleaning, baseline normalisation, and representation as raw four-second three-channel windows or channel-wise RP images. The hybrid stream retained raw ECG and RP-derived representations in parallel.

The preprocessing pipeline used the fixed ECG channel set described in Section 2.3 to generate two ECG representation branches: a raw ECG time-series branch and an RP-derived branch. Raw Shimmer ECG samples were converted from analogue-to-digital converter (ADC) units to calibrated millivolt ECG traces before ECG cleaning. The calibrated traces were cleaned using NeuroKit2 v0.2.10 [25], applying a 0.5 Hz fifth-order Butterworth high-pass filter followed by the 50 Hz powerline filtering routine. Figure 2 summarises the shared preprocessing and the downstream representation branches.

For participant *p* and ECG channel *c*, the cleaned trace $x_{p,c}\left(t\right)$ was baseline-normalised using participant/channel baseline statistics:

(1) $$z_{p,c}\left(t\right)=\frac{x_{p,c}\left(t\right)-\mu_{p,c}}{\sigma_{p,c}},$$

where $\mu_{p,c}$ and $\sigma_{p,c}$ were estimated from pre-condition resting periods for that participant and channel. This participant/channel baseline normalisation was a signal-level correction based on the corresponding pre-condition rest segment and was separate from split-dependent model-input scaling. In the raw ECG branch, task-level time series were reconstructed from the exported segment windows, divided into 4 s three-channel windows, and converted to a 2048-sample target representation using scipy.signal.resample from SciPy v1.14.0 [26]. This was a resampling step for native source lengths that varied with effective Shimmer sampling rate, rather than padding, truncation, or an unchanged pass-through. The conversion was train-independent and applied consistently across partitions. Dense raw ECG windows used a 50-sample stride between 4 s source slices, approximately 97.5% overlap at the 512 Hz target representation, yielding 56,274 retained windows (Control, N0, N1, and N2: 14,298, 14,298, 14,298, and 13,380), corresponding to 198–215 windows per retained block.

The RP-derived branch used a branch-specific sequence protocol to re-express the same baseline-normalised ECG waveform as recurrence relationships in delay-embedded state space. It used the same retained ECG channels as the raw ECG branch and did not add a separate sensor stream. Baseline-normalised 400-sample ECG frames with stride 50 were clipped to [−5,5], scaled to [0,1], and converted to binary RP images [18,19]. For channel *c*, each frame was embedded into delayed state vectors

(2) $$y_{i}^{\left(c\right)}={\left[u_{i}^{\left(c\right)},u_{i+\tau }^{\left(c\right)},\ldots ,u_{i+(d-1)\tau }^{\left(c\right)}\right]}^{⊤},$$

where $u^{\left(c\right)}$ denotes the clipped and scaled ECG frame, τ=2, and d=3. For each modelling split *f*, the recurrence threshold was defined by a train-only percentile rule:

(3) $$\epsilon_{f}=Q_{0.10}\left(\left\{{∥y_{i}^{\left(c\right)}-y_{j}^{\left(c\right)}∥}_{2}:c\in L,y_{i}^{\left(c\right)},y_{j}^{\left(c\right)}\in T_{f}\right\}\right),$$

where L denotes the retained ECG channels, $T_{f}$ denotes the training frames for split *f*, and $Q_{0.10}$ is the 10th percentile. The threshold was estimated from training data only and was not treated as a fixed global value. The binary recurrence matrix was then defined as

(4) $$R_{i,j}^{\left(c\right)}=\Theta \left(\epsilon_{f}-{∥y_{i}^{\left(c\right)}-y_{j}^{\left(c\right)}∥}_{2}\right),$$

where Θ(·) is the Heaviside step function. The selected RP representation used 400-sample frames, stride 50, ten frames per sequence, delay 2, embedding dimension 3, the fold-training-only 10th-percentile recurrence-threshold rule in Equation (3), native 396×396 recurrence matrices, and 224×224 model-side resizing. These settings were fixed for the selected representation protocol and were not tuned on held-out leave-one-round-out (LOTO) data. Supplementary Table S1 provides a bounded screening context for non-selected image-encoding and RP-derived sequence routes, but it was not an exhaustive RP parameter search. For frame *k*, let $R_{k}^{\left(c\right)}$ denote the binary recurrence matrix for channel *c*. The LA–RA, LL–RA, and Vx–RL recurrence plots were stacked as ECG-derived image planes:

(5) $$I_{k}^{\mathrm{RP}}=R_{224}\left(\left[R_{k}^{\left(\mathrm{LA}-\mathrm{RA}\right)},R_{k}^{\left(\mathrm{LL}-\mathrm{RA}\right)},R_{k}^{\left(\mathrm{Vx}-\mathrm{RL}\right)}\right]\right)\in {\left[0,1\right]}^{224\times 224\times 3},$$

where $R_{224}$ denotes resizing to 224×224 using bilinear interpolation with antialiasing and no crop. This three-channel image forms the RP-derived representation for frame *k*, and ten-frame RP sequences were formed with a sequence stride of 1. The three analysed ECG channels were stacked as image planes to preserve channel-specific recurrence structure while keeping the RP-derived input aligned with the three-channel raw ECG representation.

**Figure 3:**
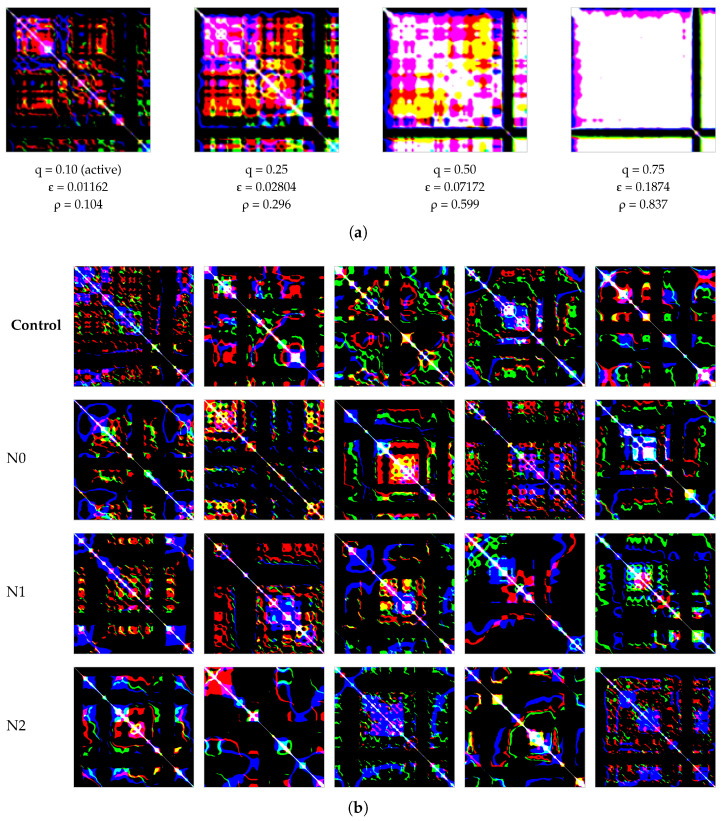
RP-derived ECG representation. (**a**) Threshold-percentile sensitivity for one retained frame. (**b**) Representative images by condition. RGB encodes LA–RA, LL–RA and Vx–RL, respectively. Mixed colours indicate channel overlap.

For the RP-derived modelling dataset, baseline-normalised ECG frames were grouped into ten-frame sequences with a sequence stride of 1. Each sequence inherited the condition label and partition of its parent and retained the task block. For each LOTO fold, RP generation yielded 64,056 frames and 61,644 fold-specific ten-frame sequence rows. Across the four folds, the held-out RP sequence rows totalled 61,644, whereas the fold-specific training RP sequence rows totalled 184,932. These RP sequence rows were modelling inputs rather than independent statistical samples, and sequence-level outputs were reduced to block-level evidence before metric computation, as described in Section 2.6 and Section 2.11. Figure 3 illustrates the recurrence-threshold choice and representative RP-derived inputs. In Figure 3a, the displayed threshold values were computed from the development-training-set frame-distance distribution for visualisation only. In the modelling pipeline, the active representation used the training-only q=0.10 rule, with $\epsilon_{f}$ recomputed separately within each model-development split or LOTO fold from that split or fold’s training-frame distances, as defined in Equation (3). Figure 3b shows representative three-channel RP-derived images from retained ECG modelling blocks. Rows correspond to Control, N0, N1, and N2, and columns show five separate illustrative retained blocks per condition. The examples illustrate branch inputs and were not selected by subject identity, round, model correctness, or performance.

The hybrid representation retained the raw ECG time-series stream and the RP-derived stream as parallel representation routes. This preserved the distinct preprocessing assumptions of each branch: RP clipping, scaling, thresholding, resizing, and sequence construction were applied only to the RP-derived stream and not to the raw ECG time-series input. The branches were therefore compared as ECG representation routes rather than as matched-complexity preprocessing pipelines.

Leakage control separated fixed signal transformations from split-dependent parameter estimation. Calibration to millivolts, ECG cleaning, baseline normalisation from the corresponding pre-condition rest segment, deterministic resampling, clipping, fixed-range scaling, window extraction, resizing, and sequence construction followed pre-specified rules applied consistently across partitions. Parameters estimated from data were nested within each model-development split or LOTO fold. For the raw ECG branch, channel normalisation and model-input scaling used training data only. For the RP-derived branch, recurrence thresholds were estimated from training frames only. For the HR/HRV-RF comparator described in Section 2.7, channel-quality selection and median imputation used training blocks only. Generated raw ECG windows and RP sequences inherited the partition of their parent task block. Boundary purging and saved zero-overlap audits were used for development partitions, and dense raw ECG windows and RP sequences were reduced to block-level scores before metric computation rather than being treated as independent result units.

### 2.6. Raw ECG, Recurrence Plot, and Hybrid Modelling Framework

The representation branches were connected to three ECG modelling streams: a raw ECG time-series model, an RP-derived model, and a hybrid model that combined both score streams. Where four-class scores were used, the scores followed the class order Control, N0, N1, and N2. In the locked scoring route, endpoint projection was treated as a downstream grouping operation, as defined in Section 2.8, rather than as a separate endpoint-specific feature extraction procedure.

The raw ECG time-series model used a MultiRocket feature transform implemented in sktime v0.40.1 [27,28,29]. Fold-training features were scaled without mean centring using StandardScaler(with_mean=False) and classified using a Ridge classifier implemented with scikit-learn v1.5.1 [30]. The fixed classifier protocol used α=215.443, the lsqr solver, intercept fitting, tolerance 0.001, 1000 maximum iterations, and no class weighting. This α value came from the prior raw ECG MultiRocket development configuration and was locked before held-out LOTO scoring. No additional α search was performed during or after the locked four-fold LOTO evaluation, and held-out LOTO folds were not used to select the value.

The RP-derived model used ten-frame sequences of three-channel RP images. These images were represented using frozen ConvNeXt-Tiny embeddings [31]. A gated temporal convolutional attention head was then trained on training sequences only. The RP temporal head used 40 epochs, AdamW optimisation [32], learning rate $3\times 10^{-4}$, weight decay $1\times 10^{-4}$, dropout 0.30, label smoothing 0.05, and batch size 512.

For the hybrid model, raw ECG and RP-derived block-level four-class score vectors were combined by fixed score-level late fusion. These vectors were used for class ranking and endpoint grouping. For input block *i*, let $s_{i}^{\mathrm{raw}}$ and $s_{i}^{\mathrm{RP}}$ denote the raw ECG and RP-derived four-class score vectors. The fused score vector before row normalisation was

(6) $$s_{i}^{\mathrm{hyb}}=0.75\;s_{i}^{\mathrm{raw}}+0.25\;s_{i}^{\mathrm{RP}}.$$

The fused vector was row-normalised before prediction. This was score-level fusion rather than feature-level fusion, and the fixed weights were not tuned on held-out evaluation folds.

For the locked held-out-round scoring route, dense outputs were reduced to block-level evidence before fusion or endpoint projection. In the raw ECG stream, the MultiRocket–Ridge classifier was refit within each model-development split or LOTO fold using the corresponding training partition and the locked protocol. Dense 4 s window outputs were converted to softmax-normalised four-class decision-score vectors by applying a deterministic row-wise softmax transform to the Ridge decision–function outputs. This transform did not fit a separate calibration model using validation, development–test, or held-out LOTO data, and the resulting vectors were used for ranking and endpoint grouping rather than as calibrated probabilities. Raw ECG scores were then grouped by retained task block, median-pooled class-wise across windows, and row-normalised. In the RP-derived stream, softmax-normalised four-class decision-score vectors from the fold-specific temporal head were grouped by retained task block, median-pooled class-wise across RP sequences, and row-normalised. This produced one raw ECG and one RP-derived block-level four-class score vector per retained task block, with dense raw ECG windows and RP sequences treated as correlated modelling instances within their parent blocks rather than independent result units.

In the locked scoring route, fusion was performed on four-class score vectors before endpoint grouping. Four-class predictions were obtained from the maximum class score. Binary and three-level endpoint predictions were obtained by grouping the four-class scores according to the endpoint hierarchy before selecting the maximum grouped score. Endpoint-specific model-development variants were evaluated separately for protocol selection, but they did not define the locked held-out-round projection route. Table 2 summarises the modelling streams.

### 2.7. Traditional HR/HRV Feature Comparator

To provide a conventional ECG-derived comparator, an HR/time-domain HRV Random Forest baseline was evaluated alongside the raw ECG, RP-derived, and hybrid branches [14,15,16]. This comparator used the same 268 retained task blocks as the ECG representation branches, but generated one HR/time-domain HRV feature vector and one block-level prediction per retained task block rather than dense windows or RP sequences. It was evaluated under the same four-fold LOTO folds and endpoint hierarchy and was included as a comparator rather than as a physiological reference standard. Using standard short-block HR/time-domain HRV feature names, features summarised short-block heart-rate and time-domain heart-rate-variability behaviour, including heart-rate summaries, NN-interval summaries, SDNN, RMSSD, pNN20, pNN50, valid NN count, peak count, artefact ratio, finite fraction, and detector or duration flags. Frequency-domain HRV features, including LF/HF, were not used as primary features because the retained intervals were short.

For each fold, unsupervised channel-quality selection and median imputation used training blocks only and did not use held-out labels, held-out predictions, or held-out model performance. The Random Forest used 1000 trees, square-root feature sampling, minimum leaf size 2, bootstrap sampling enabled, no class weighting, and fixed random state 20260623. Four-class Random Forest predict-proba scores were projected to the endpoint hierarchy using the same predefined grouping rules as the main ECG branches, and these scores were not treated as calibrated probabilities.

### 2.8. Endpoint Hierarchy and Label Construction

Where model outputs were represented as condition-level score vectors, they were projected onto a fixed endpoint hierarchy defined from the experimental workload-condition structure. The experimental manipulation defined four condition labels: Control denoted peg transfer only, whereas N0, N1, and N2 denoted peg transfer with increasing auditory N-back demand. SURG-TLX, N-back accuracy, and peg transfer summaries were used to assess whether the designed conditions followed the expected perceived-workload and task-difficulty gradient. Because workload assessment usually relies on converging subjective, behavioural, and physiological indicators rather than a single definitive physiological label, the endpoints were treated as condition-derived classification targets [5,6]. They were not physiological ground-truth labels. ECG model performance quantified the classification of fixed, condition-derived endpoint labels from ECG representations and did not establish the endpoints as latent physiological workload states.

Table 3 defines the endpoint hierarchy. The four-class condition reference preserved the original Control, N0, N1, and N2 conditions. The primary low/high endpoint grouped Control with N0 and N1 with N2 to reflect the main practical distinction between lower and higher workload states. Two secondary three-level endpoints retained alternative intermediate-condition structures. The primary low/high endpoint was defined from the designed lower-demand and higher-demand condition grouping and was interpreted as SURG-TLX-aligned because the subjective workload pattern supported this main contrast. The four-class endpoint retained the condition reference, and the three-level endpoints were secondary endpoint-boundary probes. The reported endpoint definitions were fixed for the manuscript analyses and were not redefined based on held-out LOTO performance.

Let C denote the four condition classes and let $G=\{G_{1},\ldots ,G_{K}\}$ denote an endpoint grouping. For group $G_{g}\in G$, grouped score projection was performed as

(7) $$S_{i,g}=\sum_{c\in G_{g}}s_{i,c},$$

where $s_{i,c}$ is the four-class score for block *i* and condition class *c*. The predicted endpoint label was the group with the largest grouped score.

This projection was a predefined score-grouping operation: it summed four-class score evidence within each endpoint group and was not interpreted as a linear physiological workload scale.

### 2.9. Model Development and Protocol Selection

The model-development comparison was used for protocol selection before locked evaluation. Its purpose was to compare ECG representation streams, endpoint definitions, and fusion strategies under a fixed development protocol. Because participants were represented across development partitions, this split supported within-cohort protocol selection rather than subject-independent validation. It was used to select the manuscript reporting protocol and was not treated as a standalone final generalisation claim.

The model-development analyses used a fixed block-level train, validation, and matched development–test split, with all retained modelling participants represented across the development partitions. The split contained 134 training block rows, 67 validation block rows, and 67 held-out development–test block rows. Validation macro-F1 was used for model-development monitoring and protocol selection. Matched development–test reporting was then performed after validation selection, so the matched test set reported candidate protocols retained after development monitoring rather than guiding selection among unselected variants. Because this development split was within-cohort and block-level, participant identity was not used as a held-out factor at this stage, and development–test results were interpreted as within-cohort protocol-selection diagnostics. This kept protocol selection evidence separate from the locked evaluation described in Section 2.10.

For the model-development comparison, the leakage controls described in Section 2.5 tied generated raw ECG windows and RP sequences to parent task blocks and kept validation and development–test rows out of split-dependent preprocessing estimates. For development partitions, boundary purging and saved zero-overlap audits checked that exact sequences, raw frame intervals, and raw sequence intervals were not shared across training, validation, and development–test partitions. The development split was therefore used as a controlled model-development comparison rather than as the final held-out performance estimate.

The model-development low/high comparison used an endpoint-specific aggregation route for protocol selection. In this route, dense low/high endpoint scores were summarised within the model-development pipeline before the development-stage comparison. This route was kept separate from the locked held-out-round evaluation. In the locked evaluation, dense outputs were first median-pooled within retained task blocks to form four-class block-level score vectors. Raw ECG and RP-derived scores were then combined using fixed score-level fusion, and endpoint scores were obtained by applying the predefined endpoint grouping rules.

### 2.10. Four-Fold Leave-One-Round-Out Evaluation

The locked evaluation was the four-fold LOTO evaluation. A round denotes the same repetition index across the retained Control, N0, N1, and N2 task-condition blocks. In each fold, one complete experimental round was held out across all task conditions, where retained data support allowed, while the remaining rounds from the same participants were used for training. Fold 1 held out round 1, fold 2 held out round 2, fold 3 held out round 3, and fold 4 held out round 4. P020 was retained in the modelling cohort for Control, N0, and N1 but contributed no retained N2 blocks because of the documented ShimmerECG timestamp gap. Each fold contained Control 17, N0 17, N1 17, and N2 16 block-level predictions.

Algorithm 1 summarises the score fusion and endpoint projection procedure used after block-level four-class scores were obtained in the four-fold LOTO evaluation.
**Algorithm 1:** Four-class score fusion and endpoint projection for the four-fold LOTO evaluation**Input**Block set B, stream mode *m*, block-level score vectors
$s_{b}^{\mathrm{raw}}$ and $s_{b}^{\mathrm{RP}}$, class set
C, endpoint groups $G=\{G_{1},\ldots ,G_{K}\}$, and weights $w_{\mathrm{raw}}=0.75$, $w_{\mathrm{RP}}=0.25$.**Output**Four-class labels, grouped endpoint scores, and endpoint labels for all held-out blocks.**Step 1**For each held-out block b∈B, select the branch score vector:
$$s_{b}=\left\{\begin{array}{cc} s_{b}^{\mathrm{raw}}, & m=\mathrm{raw},\\ s_{b}^{\mathrm{RP}}, & m=\mathrm{RP},\\ \mathrm{norm}\;\left(0.75\;s_{b}^{\mathrm{raw}}+0.25\;s_{b}^{\mathrm{RP}}\right), & m=\mathrm{hybrid}, \end{array}\right.$$
where norm(·) denotes row normalisation.**Step 2**Assign the four-class prediction:
${\hat{y}}_{b}^{\left(4\right)}\leftarrow \arg\max_{c\in C}s_{b,c}.$**Step 3**Project four-class scores to endpoint groups:
$$z_{b,k}\leftarrow \sum_{c\in G_{k}}s_{b,c},\;k=1,\ldots ,K,$$
and assign the grouped endpoint prediction:
$${\hat{y}}_{b}^{\left(G\right)}\leftarrow \arg\max_{k}z_{b,k}.$$**Step 4**Store ${\hat{y}}_{b}^{\left(4\right)}$, $z_{b}=\left(z_{b,1},\ldots ,z_{b,K}\right)$, and ${\hat{y}}_{b}^{\left(G\right)}$ for metric computation.

This design evaluates calibrated-user, known-participant, within-participant held-out-round performance. The same participants contributed data to training and held-out folds, so the four-fold LOTO evaluation is not a test of subject-independent generalisation, transfer to previously unseen surgeons, or deployment validation. Each held-out fold contained 67 block-level predictions after retained-data exclusions, giving 268 pooled held-out block predictions across the four folds. These are retained task-block predictions, not independent participant-level samples.

All evaluation-specific preprocessing statistics were estimated from training data only within each fold. This included raw ECG channel normalisation and recurrence-threshold estimation for the RP-derived branch.

### 2.11. Performance Metrics and Reporting

Performance was reported using accuracy, macro-F1, and balanced accuracy. All model metrics were computed at the task-block level after dense window-level and sequence-level outputs had been reduced to block-level predictions, rather than at the dense-instance level. For *N* evaluated task-block predictions and *K* endpoint classes, the reported metrics were defined as

(8) $$\begin{array}{cc} \mathrm{Accuracy} & =\frac{1}{N}\sum_{i=1}^{N}1\left({\hat{y}}_{i}=y_{i}\right),\\ \mathrm{Balanced}\;\mathrm{accuracy} & =\frac{1}{K}\sum_{k=1}^{K}{\mathrm{Recall}}_{k},\\ \mathrm{Macro}\text{-}F1 & =\frac{1}{K}\sum_{k=1}^{K}F1_{k}. \end{array}$$

Macro-F1 was used because the endpoint hierarchy includes binary, three-level, and four-class endpoints with different class structures. Balanced accuracy provided a class-balanced interpretation within each endpoint definition. Because class number and class prevalence differ across the binary, three-level, and four-class tasks, metrics were interpreted within endpoint definitions rather than as direct cross-endpoint difficulty comparisons.

Uncertainty for four-fold LOTO metrics was summarised using participant-clustered percentile bootstrap 95 percent confidence intervals, with participant as the cluster unit. Each bootstrap resample drew 17 participant clusters with replacement and retained all held-out blocks for each sampled participant, using 10,000 resamples. Paired branch-difference intervals used the same resampled participant clusters and their retained blocks.

Planned paired branch comparisons used participant-clustered randomisation tests with participant as the exchange unit. For each planned comparison, exact enumeration was performed over $2^{17}$ participant-level branch swaps, preserving all retained blocks within each participant. Holm correction was applied within the planned primary low/high macro-F1 and balanced-accuracy comparison families. Branch differences were interpreted using participant-clustered confidence intervals and the planned paired tests. Differences were described as supported when the planned paired test supported them and as numerical when that support was not present. The full all-endpoint test grid was treated as exploratory Supplementary Table S2 material.

Model-development metrics and four-fold LOTO metrics were reported separately. Development metrics were used to monitor validation macro-F1 and to report matched development–test performance after protocol selection. The primary scientific comparison used the locked four-fold LOTO primary low/high endpoint, while model-development metrics supported protocol selection and diagnostic comparison. The primary four-fold LOTO aggregate metrics were computed from pooled held-out task-block predictions across the four folds. Fold-wise metrics were retained as diagnostic summaries. Confusion matrices in the Results were computed from pooled held-out task-block predictions across LOTO folds unless otherwise stated, and were used to describe endpoint-specific error structure.

## 3. Results

### 3.1. Subjective Workload and Endpoint Rationale

SURG-TLX characterised perceived workload alignment for the endpoint rationale in the ECG modelling cohort (Table 4). Total SURG-TLX scores differed across Control, N0, N1, and N2, F(3,48)=34.947, $p=3.98\times 10^{-12}$, with partial $\eta^{2}=0.686$. The planned low/high endpoint contrasted the participant-level mean of Control plus N0 with the participant-level mean of N1 plus N2. The low and high groupings had mean (standard deviation, SD) SURG-TLX scores of 44.25 (13.28) and 68.26 (11.14), respectively, giving a mean paired difference of 24.02 points (95% confidence interval (CI) 19.44–28.60), t(16)=11.13, $p=6.10\times 10^{-9}$, Cohen’s $d_{z}=2.70$. Effect-size reporting in the main text focused on this planned endpoint-rationale contrast. Assumption checks supported the same interpretation: Mauchly’s test did not reject sphericity (p=0.502), Greenhouse–Geisser correction was not required, the planned high-minus-low paired differences showed no evidence against normality by Shapiro–Wilk testing (p=0.945), and a Wilcoxon signed-rank sensitivity analysis supported the planned low/high contrast ($p=1.53\times 10^{-5}$).

**Figure 4:**
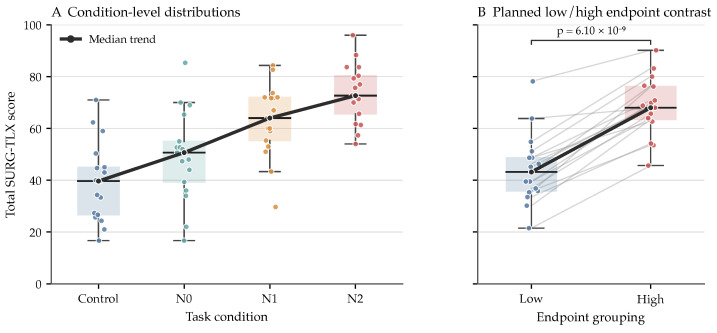
SURG-TLX endpoint-rationale analysis in the ECG modelling cohort. (**A**) Total SURG-TLX by condition with participant observations and median trend. (**B**) Planned low/high contrast, with Low = Control plus N0 and High = N1 plus N2 at the participant level.

Figure 4 shows the condition-level subjective workload pattern together with the planned Control plus N0 versus N1 plus N2 endpoint contrast.

Task-performance measures provided descriptive manipulation-check evidence and were not used as model labels. N-back accuracy was analysed within the N0, N1, and N2 conditions and was interpreted with caution because N0 showed ceiling behaviour. Mauchly’s test did not support sphericity for N-back accuracy (p=0.00155), so the repeated-measures effect across N0, N1, and N2 was interpreted using the Greenhouse–Geisser correction, F(1.27,20.28)=25.527, $p=2.21\times 10^{-5}$. A Friedman sensitivity analysis was consistent with a condition effect across N0, N1, and N2 ($p=1.38\times 10^{-7}$). N-back accuracy was highest in N0 and lowest in N2, while peg transfer summaries showed the lowest mean number of pegs transferred and the highest failure-to-transfer counts in N2. Together, these subjective and task-performance patterns followed the expected perceived workload and task-difficulty gradient across the designed conditions. Additional adjacent and endpoint-aligned SURG-TLX contrasts were treated as supporting analyses rather than as the basis for endpoint construction.

### 3.2. Model-Development Comparison Across Branches and Endpoints

The model-development split served as development-stage protocol selection and diagnostic evidence before the locked four-fold LOTO evaluation. Validation macro-F1 was the monitoring criterion during this stage. Matched development–test metrics, then summarised retained candidate protocols after validation monitoring, rather than providing final generalisation evidence. Table 5 reports this harmonised validation and matched development–test grid, where raw ECG, RP-derived, and hybrid branches are compared using the same block-level four-class score-projection route across the four endpoint definitions.

Within the matched development–test part of the harmonised grid, the hybrid branch had higher macro-F1 than the raw ECG branch for the four-class condition reference by 0.028 and for the three-level endpoint A by 0.035, matched the raw ECG on the primary low/high endpoint, and was lower than the raw ECG on the three-level endpoint B by 0.056. These differences were used as development-stage descriptive evidence. The RP-derived branch was generally lower than the raw ECG and hybrid branches, but it remained informative as a representation comparator. A separate endpoint-first low/high route documented the development-stage path used for low/high protocol selection and is summarised in Supplementary Table S1. It reported validation macro-F1 and balanced accuracy of 0.925 and 0.926, and matched development–test macro-F1 and balanced accuracy of 0.881 and 0.881. This route was kept separate from the harmonised grid because it used endpoint-specific score grouping rather than the branch-comparable four-class score-projection route.

**Figure 5:**
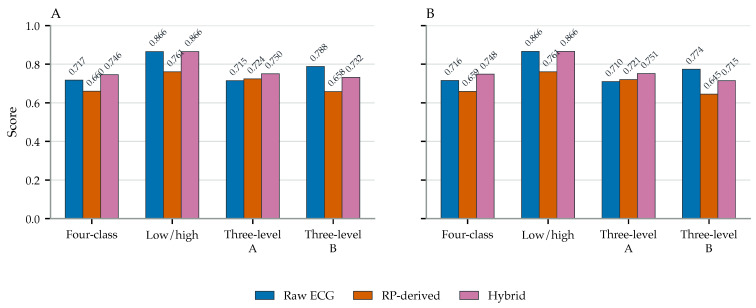
Model-development branch-by-endpoint performance. (**A**) Macro-F1. (**B**) Balanced accuracy. Bars show matched development–test macro-F1 and balanced accuracy for raw ECG, RP-derived, and hybrid branches across endpoint definitions. Exact values are reported in Table 5.

Figure 5 visualises the model-development branch-by-endpoint pattern, complementing the exact values in Table 5.

### 3.3. Four-Fold Leave-One-Round-Out Evaluation Across Branches and Endpoints

The four-fold LOTO evaluation was the locked held-out-round evaluation. It used held-out task rounds from known participants, with metrics computed from pooled held-out task-block predictions across the four folds. Each fold contained 67 held-out blocks, and the pooled evaluation contained 268 held-out task-block predictions. Table 6 reports the descriptive branch-by-endpoint performance matrix for this calibrated-user within-participant evaluation. Primary low/high branch comparisons were performed with participant-clustered paired tests on held-out task-block predictions. Fold-wise metrics are reported later as diagnostic summaries.

Table 6 provides the all-endpoint descriptive matrix. Raw ECG had the highest point estimates for the four-class condition reference and the primary low/high endpoint. The hybrid branch remained close to raw ECG on the primary low/high endpoint and had higher point estimates than raw ECG for both secondary three-level endpoints. RP-derived macro-F1 was lower than raw ECG and hybrid across the four endpoints. The HRV-RF comparator was evaluated on the same retained block IDs and endpoint hierarchy, but it is reported as a conventional ECG-derived feature comparator rather than as an ECG representation branch. Its point estimates were lower than the representation branches across endpoints.

Table 7 reports participant-clustered uncertainty and planned paired comparisons for the primary low/high endpoint. Raw ECG was numerically higher than hybrid in macro-F1 and balanced accuracy, but the paired confidence intervals included zero and the planned paired tests did not support a raw ECG versus hybrid difference after Holm correction. For both macro-F1 and balanced accuracy, the planned paired tests supported higher raw ECG and hybrid performance than RP-derived and HRV-RF on the primary low/high endpoint. The remaining pairwise entries in Table 7 show the planned comparison family and support transparency of the primary endpoint reporting.

**Figure 6:**
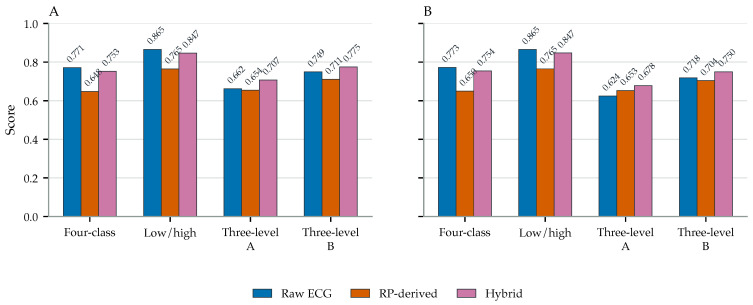
Four-fold LOTO branch-by-endpoint performance. (**A**) Macro-F1. (**B**) Balanced accuracy. Bars show macro-F1 and balanced accuracy from pooled held-out task-block predictions for raw ECG, RP-derived, and hybrid branches. Exact values are reported in Table 6.

Figure 6 visualises the three ECG representation branches in the four-fold LOTO evaluation. The HRV-RF comparator is reported in Table 6 and Table 7 and Supplementary Table S2.

### 3.4. Representation Complementarity and Endpoint-Dependent Behaviour

The development and LOTO results showed endpoint-dependent branch behaviour. In the matched development–test part of the model-development split, the harmonised hybrid branch had higher macro-F1 than raw ECG for the four-class condition reference by 0.028 and for the three-level endpoint A by 0.035, matched the raw ECG on the primary low/high endpoint, and was lower for the three-level endpoint B by 0.056. These development-stage differences were descriptive protocol-selection evidence.

In the four-fold LOTO evaluation, raw ECG was numerically stronger than hybrid for the four-class condition reference and primary low/high endpoint, with hybrid-minus-raw macro-F1 deltas of −0.019 and −0.018, respectively. For the primary low/high endpoint, Table 7 showed that the raw ECG versus hybrid difference was only numerical. Hybrid had higher point estimates than raw ECG for the secondary three-level endpoints, with hybrid-minus-raw macro-F1 deltas of 0.045 for three-level endpoint A and 0.026 for three-level endpoint B. Because planned paired testing focused on the primary low/high endpoint, these secondary endpoint patterns were interpreted as exploratory endpoint-boundary behaviour rather than model-selection evidence. Supplementary Table S2 reports all-endpoint participant-clustered uncertainty, pairwise CI summaries, and the exploratory paired-test grid.

### 3.5. Error Structure, Fold-Wise Diagnostics, and Supplementary Context

**Figure 7:**
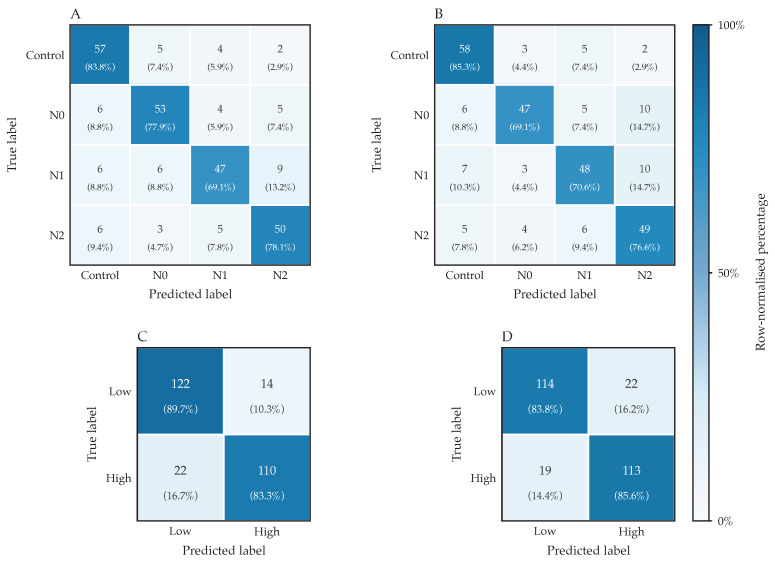
Selected confusion matrices from the four-fold LOTO evaluation. (**A**) Raw ECG, four-class. (**B**) Hybrid, four-class. (**C**) Raw ECG, low/high. (**D**) Hybrid, low/high. Cells show held-out task-block counts and row-normalised percentages.

Fold-wise and error-structure diagnostics were retained to summarise variation across held-out rounds and endpoint boundaries. Table 8 reports fold-wise macro-F1 for each held-out round across branches and endpoints. This compact diagnostic table reports macro-F1 only, while aggregate accuracy and balanced accuracy are reported in Table 6 and Supplementary Table S2. Formal branch-comparison summaries for the primary low/high endpoint are reported in Table 7, and the full all-endpoint uncertainty and exploratory paired-comparison grid is reported in Supplementary Table S2. Figure 7 shows selected raw ECG and hybrid confusion matrices for the four-class condition reference and the primary low/high endpoint.

The selected confusion matrices describe endpoint-specific error structure. In the four-class condition reference, off-diagonal errors were distributed across multiple task-condition labels rather than forming a single uniform failure mode. Adjacent-condition errors, including N1–N2 cross-confusions, were visible in the displayed matrices. In the primary low/high endpoint, the off-diagonal cells summarised cross-boundary mistakes between the two endpoint groups. These matrices were used as descriptive diagnostics rather than as separate inferential tests. Supplementary Table S1 summarises benchmark and ablation comparisons that contextualise protocol selection and representation choices without serving as primary evaluation claims. Supplementary Table S2 provides the all-endpoint uncertainty and exploratory paired-comparison material supporting the compact Results display.

## 4. Discussion

### 4.1. Principal Findings

This study supports a lower-burden ECG-only route for block-level classification of condition-derived workload states in structured laparoscopic training. The clearest evidence came from the planned primary low/high endpoint. Raw ECG had the highest point estimate on this endpoint, and both raw ECG and hybrid score-level fusion were supported over RP-derived and HRV-RF under the planned paired tests. The raw ECG versus hybrid difference remained numerical only.

These findings address the study objectives. ECG-only representations classified broad condition-derived workload states in the controlled peg transfer and auditory N-back setting. The comparison across raw ECG, RP-derived, hybrid, and HRV-RF routes showed endpoint-dependent representation behaviour. Raw ECG was the strongest broad-boundary route in this dataset, while hybrid score-level fusion produced higher point estimates on selected secondary three-level endpoints. The known-participant four-fold LOTO evaluation provided calibrated-user held-out-round evidence for repeated structured training, with inference centred on task-block predictions rather than dense intermediate inputs.

SURG-TLX and task-performance summaries supported the workload manipulation and endpoint rationale but were not model inputs. The classifiers used ECG to predict fixed, condition-derived endpoint labels. The findings therefore support ECG-based classification of experimentally structured workload states, interpreted alongside subjective and behavioural alignment without treating the outputs as direct measurements of latent CWL.

### 4.2. Endpoint-Aware Interpretation of ECG Representation Strategies

The primary low/high endpoint matched the main planned contrast in the task design and SURG-TLX pattern. It grouped Control and N0 as the lower-demand side and N1 and N2 as the higher-demand side, which reduced adjacent-condition ambiguity. This framing is consistent with stronger low/high classification than the four-class condition reference and the secondary three-level endpoints.

The four-class and three-level endpoints served different interpretive roles. The four-class endpoint retained the original Control, N0, N1, and N2 task structure, which made it useful for examining condition-level separability. The two three-level endpoints examined how branch behaviour changed when one intermediate condition was grouped with the lower or higher side. These views added diagnostic granularity, but they also introduced harder adjacent boundaries.

This distinction matters for a low-burden ECG-only route. Psychophysiological workload studies treat subjective, behavioural, and physiological evidence as complementary, and physiological-signal machine-learning studies vary substantially in signals, labels, representations, and validation schemes [5,6,17]. For broad workload-state separation in repeated structured training, the low/high results suggest that an ECG-only route can provide useful physiological classification evidence. For finer condition-level boundaries, lower performance and shifting branch rankings suggest a harder adjacent-boundary problem for ECG alone, especially when labels combine workload-related demand with task-condition structure. The intended endpoint, therefore, determines the practical question being asked of the sensing route.

Raw ECG retained the strongest evidence for the broad low/high endpoint. The hybrid branch showed higher point estimates on selected secondary endpoints, which is compatible with possible complementarity between raw ECG waveform structure and RP-derived recurrence structure. The practical contribution of RP-derived information remains unresolved because the RP-derived branch alone was lower than raw ECG and hybrid on the primary endpoint, and the hybrid branch did not consistently outperform raw ECG across endpoints. Supplementary Table S1 provides bounded benchmark and ablation context, but it does not isolate which representation, fusion, preprocessing, or classifier-design choices drove these endpoint-specific patterns. Accordingly, Supplementary Table S1 should be read as bounded design-context evidence rather than as a complete factorial component-ablation study.

### 4.3. Model Development and Held-Out-Round Evaluation

On the primary low/high endpoint, raw ECG and hybrid remained close by point estimate (macro-F1 0.865 and 0.847), and the raw ECG–hybrid paired difference was numerical only. Fold-wise low/high macro-F1 ranges for raw ECG and hybrid-supported diagnostic interpretation of round-to-round variation without treating the four folds as independent validation contexts.

For the primary endpoint in this dataset, the waveform-level representation routes achieved stronger discrimination than the short-block HR/time-domain HRV comparator. This interpretation is limited to the current endpoint, cohort, and evaluation protocol. It does not imply that HRV features are generally inadequate or that the traditional ECG-feature design space has been exhausted.

The model-development split and the four-fold LOTO evaluation contribute different evidence. The development stage supported protocol selection and branch-comparable diagnostics. The locked four-fold LOTO evaluation then tested held-out task rounds from known participants using the selected evaluation route. This separation helps distinguish development-stage representation behaviour from the primary held-out-round evidence.

The LOTO design is relevant to calibrated structured training because such training often involves repeated attempts by known trainees or surgeons under changing task demands [2,21]. Holding out one round tests whether the selected route carries across retained rounds from the same participant cohort. This supports the repeated-training use case addressed here. Broader generalisation remains a separate validation question.

### 4.4. Practical Implications for Low-Burden ECG-Only Training Assessment

The practical implication of the ECG-only route is reduced instrumentation burden within a calibrated training workflow. In the present study, ECG setup was completed as part of participant preparation in approximately 20 min, including taking the participant to a private room and applying the ECG electrodes. No ECG-related discomfort concerns were documented during routine study conduct. These observations support the feasibility of ECG-only sensing in this controlled training workflow, although they were not collected as formal human-factors outcomes.

The ECG-only workflow should be viewed against prior MAESTRO multimodal sensing workflows. An earlier MAESTRO pilot in laparoscopic peg transfer used a multimodal sensor network including fNIRS, EEG, eye tracking, photoplethysmography (PPG), galvanic skin response (GSR), ECG, EMG, and skin temperature, with sensor fitting and calibration before task performance [33]. A later simulated laparoscopic cholecystectomy study recorded EEG, fNIRS, EMG, ECG, and pupil-diameter measures as part of a MAESTRO multimodal CWL platform [9]. Compared with these denser workflows, the present ECG-only route uses fewer worn sensing components and is therefore a plausible lower-burden option for repeated structured training. This is a practical design inference rather than evidence that ECG-only sensing is superior to multimodal assessment.

The immediate educational implication is calibrated assessment within structured training. ECG-only block-level classification could complement SURG-TLX and task-performance summaries by adding a physiological signal route during repeated task attempts. The current evidence is not a real-time monitoring system and should not be used to infer clinical readiness. Its value is in clarifying what can be learned from ECG-only data under a controlled task design, a fixed endpoint hierarchy, and leakage-aware held-out-round evaluation.

### 4.5. Limitations

Several limitations define the scope of the findings. First, the study used a controlled laparoscopic training task and a limited ECG modelling cohort. Participant variability, cohort specificity, and the P020 N2 timestamp exclusion remain important context for interpretation. The results should therefore be read as evidence for the retained cohort and task context rather than broad surgical generalisation.

Second, the labels were condition-derived. They were aligned with task design, SURG-TLX, and manipulation-check evidence, but they were not direct measurements of latent physiological CWL. The models may therefore capture task-condition structure as well as workload-related physiological variation. None of the subjective, behavioural, or ECG-derived measures should be treated as physiological ground truth. The study also did not include a quantitative endpoint-separability analysis linking SURG-TLX gradients to inter-class distance, confusion entropy, or representation-space separation.

Third, the evaluation was calibrated to each user and conducted on known participants. Four-fold LOTO tested held-out rounds from the same participant cohort. This is a scope limitation of the current evaluation rather than evidence that the route would fail under broader validation. The study did not test leave-one-subject-out (LOSO) generalisation to unseen participants. The participant-clustered analyses improve uncertainty reporting within this scope but do not change the validation setting.

Fourth, all model metrics were reported at the task-block level after dense raw ECG windows and RP sequences were reduced to block-level predictions. This avoided treating dense intermediate inputs as independent endpoint evidence. It also means that within-block temporal dynamics, continuous workload tracking, and prospective real-time monitoring were outside the present evaluation.

Fifth, the practical burden of ECG-only sensing was not evaluated as a formal outcome. Setup time, participant comfort, workflow interference, signal-loss burden, and ergonomic impact were not measured using a predefined human-factors protocol. The approximately 20 min setup and absence of documented ECG-related discomfort concerns should therefore be interpreted as workflow observations and not formal evidence of ergonomic superiority.

Finally, mechanistic interpretation of RP-derived and hybrid behaviour remains limited. The study compared ECG representation routes and score-level fusion, but it did not establish why RP-derived structure helped or hindered particular endpoint boundaries. The RP settings and fusion strategy were bounded rather than exhaustively optimised, and the HRV-RF comparator was a short-block HR/time-domain HRV baseline rather than an exhaustive traditional-feature benchmark.

### 4.6. Future Work

Future work should proceed through methodological, validation, and endpoint-development directions. The most direct methodological extension is a structured component-ablation programme built around the present ECG representation framework. Rather than treating the RP-derived and hybrid routes as single indivisible blocks, future studies should vary recurrence encoding, fusion weighting, preprocessing and windowing choices, embedding architecture, and classifier parameters while keeping endpoint definitions and the evaluation protocol fixed. This would test which parts of the RP-derived and hybrid route add information beyond the raw ECG waveform.

Within this methodological direction, a related extension is to use richer ECG routes to support lower-complexity modelling pathways. If RP-derived or hybrid representations provide stable complementary information under future validation, knowledge distillation or teacher–student experiments could test whether hybrid or RP-informed outputs can guide compact raw ECG models. Such work could retain the low-burden ECG-only sensing route while reducing dependence on heavier image-sequence processing. Temporally resolved modelling, fuller HRV and traditional-feature baselines, endpoint-separability analyses, and representation-specific interpretability, including saliency or gradient-weighted class activation mapping (Grad-CAM) analyses where appropriate, should be developed alongside these component studies.

A second direction is validation in clinical, translational, and human-factors settings. Repeated-session surgical-training studies could test whether calibration from an initial session improves classification in later sessions from the same trainee. This design directly extends the calibrated-user setting evaluated here and may be more realistic than immediate subject-independent deployment. Larger cohorts would then support LOSO and cross-session validation to assess transfer to new users and new sessions. Future studies should also measure practical burden directly, including setup time, comfort, movement interference, signal loss, workflow compatibility, and participant experience. This would test the ergonomic and educational value of ECG-only sensing rather than inferring it from the sensing route alone.

The third direction is endpoint refinement and eventual real-time evaluation. Condition-derived labels are useful for controlled training, but future endpoints should integrate subjective workload, behavioural performance, technical skill, instructor or expert ratings, and longitudinal learning trajectories. Multimodal validation could help test when ECG-only estimates are sufficient and when additional sensing channels are needed. Once temporal models and validation protocols are more mature, real-time or near-real-time ECG-based workload assessment could be evaluated as a prospective validation question rather than inferred from offline block-level classification.

## 5. Conclusions

This study supports ECG-only block-level workload-state classification in a controlled laparoscopic training setting. The strongest evidence came from the planned primary low/high endpoint, where raw ECG had the highest point estimate and raw ECG and hybrid were supported over RP-derived and HRV-RF. The raw ECG versus hybrid difference remained numerical only. Secondary endpoints showed endpoint-dependent branch behaviour, leaving RP-derived complementarity as a targeted future question rather than a general hybrid advantage.

The main implication is that a lower-burden ECG-only route can provide ECG-derived classification evidence for structured training assessment when interpreted within its validation scope. The findings are retrospective and based on held-out rounds from known participants. They should therefore be read as calibrated-user evidence rather than LOSO or deployment validation. Future work should test larger-cohort generalisation, repeated-session calibration, practical burden and human-factors outcomes, endpoint refinement, and temporally resolved ECG modelling.

## Figures and Tables

**Table 1:** Participant characteristics for the recruited cohort and ECG modelling cohort.

Characteristic	Recruited Cohort	ECG Modelling Cohort
Participants, *n*	20	17
Sex, *n*	13 male, 7 female	10 male, 7 female
Handedness, *n*	17 right-handed, 3 left-handed	14 right-handed, 3 left-handed
Training role, *n*	16 SHO, 2 SpR, 1 FY, 1 student	14 SHO, 2 SpR, 1 student
Laparoscopic assists	50 (8.5–60), 0–300	50 (20–60), 0–300
Laparoscopic procedures	8 (0–20), 0–100	10 (0–20), 0–100

Note: IQR = interquartile range, SHO = senior house officer, SpR = specialist registrar, FY = foundation year. Laparoscopic experience variables are reported as median (IQR), range. Sleep duration and caffeine intake were pre-task state variables. In the recruited cohort, the corresponding values were 6 (5.5–7) h (range: 0–8 h) and 1 (0.75–2) cups (range: 0–4 cups), respectively. In the ECG modelling cohort, the corresponding values were 6 (6–7) h (range: 0–8 h) and 1 (0–2) cups (range: 0–4 cups), respectively.

**Table 2:** ECG modelling framework components.

Stream	Modelling Approach
Raw ECG	Four s three-channel ECG windows with 2048 samples were transformed using MultiRocket features, scaled without mean centring, and classified with a fixed-alpha Ridge classifier.
RP-derived	Ten-frame sequences of three-channel RP images were represented using frozen ConvNeXt-Tiny embeddings followed by a gated temporal convolutional attention head.
Hybrid	Raw ECG and RP-derived four-class score vectors were combined using fixed score-level late fusion with a 0.75 raw ECG weight and 0.25 RP-derived weight.

Note: RP = recurrence plot.

**Table 3:** Workload endpoint definitions used for ECG model development and evaluation.

Endpoint	Grouping	Use in This Study
Four-class condition reference	Control, N0, N1, and N2	Fine-grained condition reference for four-class classification
Primary low/high endpoint	Control plus N0 versus N1 plus N2	Primary practical endpoint for workload-state evaluation
Three-level endpoint A	Control plus N0, with N1 and N2 separate	Secondary endpoint retaining N1 and N2 as separate higher-workload conditions
Three-level endpoint B	Control and N0 separate, with N1 plus N2	Secondary endpoint retaining Control and N0 separately while grouping N1 with N2

**Table 4:** SURG-TLX Endpoint-rationale and task-performance manipulation-check summaries in the ECG modelling cohort. Values are means (SD) unless stated otherwise.

Condition	*n*	SURG-TLX Total	Pegs Transferred	Failed Transfers	N-Back Accuracy (%)
Control	17	39.1 (15.3)	13.9 (5.2)	0.1 (0.5)	Not applicable
N0	17	49.4 (17.3)	14.5 (5.3)	0.4 (0.6)	100.0 (0.0)
N1	17	63.0 (14.0)	14.2 (5.7)	0.6 (1.1)	94.1 (10.2)
N2	17	73.6 (11.3)	11.8 (5.5)	1.4 (1.7)	69.5 (23.6)

Note: Failed transfers denote failure-to-transfer peg counts. N-back accuracy was not applicable for Control by design. SURG-TLX = Surgery Task Load Index. SD = standard deviation. SURG-TLX variables supported endpoint rationale, while N-back and peg transfer variables supported manipulation checks.

**Table 5:** Model-development validation and matched development–test performance across ECG representation branches and endpoints.

Branch	Endpoint	Val. Macro-F1	Test Accuracy	Test Macro-F1	Test Balanced Acc.
Raw ECG	Four-class	0.722	0.716	0.717	0.716
	Low/high	0.910	0.866	0.866	0.866
	Three-level A	0.660	0.746	0.715	0.710
	Three-level B	0.841	0.806	0.788	0.774
RP-derived	Four-class	0.671	0.657	0.660	0.659
	Low/high	0.851	0.761	0.761	0.761
	Three-level A	0.700	0.746	0.724	0.721
	Three-level B	0.760	0.687	0.658	0.645
Hybrid	Four-class	0.748	0.746	0.746	0.748
	Low/high	0.910	0.866	0.866	0.866
	Three-level A	0.711	0.776	0.750	0.751
	Three-level B	0.858	0.761	0.732	0.715

Note: Val. = validation. Test = matched development test. acc. = accuracy. RP = recurrence plot. Validation macro-F1 was used for model-development monitoring. Rows use the harmonised branch-comparable scoring route. Selected endpoint-specific low/high development rows are discussed separately in the text.

**Table 6:** Four-fold LOTO performance across ECG representation branches and endpoints with the HRV-RF comparator.

Model	Endpoint	Accuracy	Macro-F1	Balanced Acc.
Raw ECG	Four-class	0.772	0.771	0.773
	Low/high	0.866	0.865	0.865
	Three-level A	0.720	0.662	0.624
	Three-level B	0.784	0.749	0.718
RP-derived	Four-class	0.649	0.648	0.650
	Low/high	0.765	0.765	0.765
	Three-level A	0.687	0.654	0.653
	Three-level B	0.728	0.711	0.704
Hybrid	Four-class	0.754	0.753	0.754
	Low/high	0.847	0.847	0.847
	Three-level A	0.754	0.707	0.678
	Three-level B	0.799	0.775	0.750
HRV-RF	Four-class	0.358	0.358	0.359
	Low/high	0.649	0.648	0.649
	Three-level A	0.541	0.450	0.449
	Three-level B	0.474	0.377	0.387

Note: Values are computed from 268 pooled held-out task-block predictions, with 67 blocks per fold. LOTO = leave-one-round-out. acc. = accuracy. Raw ECG, RP-derived, and hybrid are representation branches; HRV-RF is a conventional HR/time-domain heart-rate-variability Random Forest comparator. This is a calibrated-user within-participant held-out-round evaluation.

**Table 7:** Participant-clustered uncertainty and planned paired comparisons for the primary low/high endpoint in the four-fold LOTO evaluation.

**Panel A. Primary Low/High Endpoint Performance**		
**Model**	**Macro-F1 (95% CI)**	**Balanced acc. (95% CI)**
Raw ECG	0.865 (0.809–0.919)	0.865 (0.809–0.918)
Hybrid	0.847 (0.790–0.901)	0.847 (0.788–0.901)
RP-derived	0.765 (0.709–0.825)	0.765 (0.710–0.824)
HRV-RF	0.648 (0.585–0.710)	0.649 (0.588–0.710)
**Panel B. Planned Paired Primary Low/High Comparisons**		
**Comparison**	**Macro-F1 Difference, CI, Holm p, Interpretation**	**Balanced acc. Difference, CI, Holm p, Interpretation**
Raw ECG vs. Hybrid	Delta 0.018; CI -0.022 to 0.056; Holm p 0.485; numerical only	Delta 0.018; CI -0.023 to 0.055; Holm p 0.485; numerical only
Raw ECG vs. RP-derived	Delta 0.100; CI 0.052 to 0.151; Holm p 0.005; supported	Delta 0.100; CI 0.051 to 0.151; Holm p 0.005; supported
Hybrid vs. RP-derived	Delta 0.082; CI 0.051 to 0.116; Holm p 0.002; supported	Delta 0.082; CI 0.051 to 0.116; Holm p 0.002; supported
Raw ECG vs. HRV-RF	Delta 0.217; CI 0.130 to 0.309; Holm p 0.002; supported	Delta 0.217; CI 0.130 to 0.306; Holm p 0.002; supported
RP-derived vs. HRV-RF	Delta 0.117; CI 0.024 to 0.215; Holm p 0.060; nominal only after Holm	Delta 0.117; CI 0.024 to 0.214; Holm p 0.060; nominal only after Holm
Hybrid vs. HRV-RF	Delta 0.199; CI 0.117 to 0.289; Holm p 0.002; supported	Delta 0.199; CI 0.114 to 0.288; Holm p 0.002; supported

Note: CIs are participant-clustered 95% confidence intervals. Planned paired tests used exact participant-clustered randomisation with Holm correction within the primary low/high macro-F1 and balanced-accuracy families. “Supported” denotes support under the planned paired test; “numerical only” denotes no planned-test support; “nominal only after Holm” denotes nominal unadjusted evidence that did not remain supported after Holm correction.

**Table 8:** Fold-wise macro-F1 values across the four held-out rounds in the four-fold LOTO evaluation.

Branch	Endpoint	Fold 1	Fold 2	Fold 3	Fold 4	Mean (SD)
Raw ECG	Four-class	0.727	0.819	0.837	0.699	0.771 (0.067)
	Low/high	0.835	0.865	0.910	0.850	0.865 (0.032)
	Three-level A	0.695	0.599	0.700	0.641	0.659 (0.048)
	Three-level B	0.736	0.813	0.725	0.712	0.747 (0.046)
RP-derived	Four-class	0.639	0.691	0.689	0.584	0.651 (0.050)
	Low/high	0.806	0.761	0.787	0.698	0.763 (0.047)
	Three-level A	0.662	0.645	0.696	0.617	0.655 (0.033)
	Three-level B	0.705	0.743	0.745	0.648	0.710 (0.045)
Hybrid	Four-class	0.733	0.793	0.775	0.715	0.754 (0.036)
	Low/high	0.850	0.850	0.865	0.819	0.846 (0.019)
	Three-level A	0.637	0.690	0.808	0.681	0.704 (0.073)
	Three-level B	0.768	0.829	0.787	0.710	0.774 (0.049)

Note: Values are fold-wise macro-F1. Mean (SD) is computed across the four folds. SD = standard deviation. Each fold contains 67 held-out task blocks. Accuracy and balanced accuracy are omitted from this compact diagnostic table.

## Data Availability

The data presented in this study are not publicly available due to participant privacy and ethical restrictions. Data may be available from the corresponding author upon reasonable request and subject to institutional approval.

## Supplementary Materials

The following supporting information can be downloaded at: https://www.mdpi.com/article/10.3390/s26144427/s1, Supplementary Table S1: Supporting benchmark and bounded ablation-context comparisons contextualising protocol selection and ECG representation choices, and Supplementary Table S2: Uncertainty estimates and exploratory paired comparisons across workload endpoints in the four-fold LOTO evaluation.
