# Supplementary material for: ECG-Only Cognitive Workload State Classification in Laparoscopic Training Using Raw and Recurrence-Plot Representations
Source: Sensors (Basel). 2026 Jul 12;26(14):4427. doi: 10.3390/s26144427 (PMC13417929; doi:10.3390/s26144427)
Supplement: Supplementary file 1 [file sensors-26-04427-s001.zip › sensors-4364131-supplementary.pdf]

Supplementary Material

Supplementary Table S1. Supporting benchmark and bounded ablation-context comparisons contextualising protocol selection and ECG representation choices.

Comparison	Model or analysis	Metric summary	Interpretive role
Raw ECG model-development references	Rocket-family and MultiRocket raw ECG models	Validation/development-test macro-F1 0.722/0.717 in the block-level reference analysis. A separate robustness reference analysis gave test macro-F1 0.624.	Provided raw time-series context for subsequent combined models.
Hybrid four-class model-development analysis	Hybrid raw ECG and RP-derived model	Development-test macro-F1 0.761. Balanced accuracy 0.763.	Contextualised the four-class condition reference during model development.
Hybrid low/high model-development analysis	Hybrid raw ECG and RP-derived scoring for the primary low/high endpoint	Development-test macro-F1 0.881. Balanced accuracy 0.881.	Contextualised low/high endpoint protocol selection during model development.
Raw ECG four-fold LOTO evaluation	Raw ECG MultiRocket model	Low/high macro-F1 0.865. Balanced accuracy 0.865.	Contextualised raw ECG performance for the primary low/high endpoint in the four-fold LOTO evaluation.
Hybrid four-fold LOTO evaluation	Fixed 0.75/0.25 score fusion of raw ECG and RP-derived outputs	Low/high macro-F1 0.847. Three-level endpoint A macro-F1 0.707. Three-level endpoint B macro-F1 0.775.	Contextualised possible endpoint-dependent complementarity between raw ECG and RP-derived scores.
Endpoint-specific retraining	Endpoint-specific raw ECG MultiRocket and RP-derived temporal models	Development-test low/high macro-F1 was 0.866 for raw ECG and 0.752 for RP-derived.	Provided ablation context for retaining grouped-score projection rather than endpoint-specific single-stream retraining.
Single-image two-dimensional ECG encodings	Frozen ConvNeXt models using recurrence-plot, Gramian angular field, and Markov transition field inputs	Validation results were retained only as screening context.	Provided ablation context for not selecting single-image ECG encodings.
Alternative RP-derived sequence protocol	Local RP-derived sequence protocol with raw ECG margin information	Validation results were retained only as screening context.	Provided bounded ablation context for recurrence-plot sequence design. This was not an exhaustive recurrence-plot sequence search.
Score-stability post-processing	Low/high score trajectory stability screen	Validation metric unchanged relative to endpoint-grouped low/high scoring. No prediction changes.	Provided ablation context for not adding score-stability post-processing to the selected low/high endpoint model.
Raw ECG deep-learning comparator	Raw ECG deep-learning comparator models	Best low/high validation/development-test macro-F1 0.791/0.746.	Provided comparator context and did not replace the raw ECG MultiRocket model.
Full-block multiple-instance learning	Offline full-block raw ECG, RP-derived, and hybrid multiple-instance learning models	Best hybrid low/high validation/development-test macro-F1 0.700/0.581.	Provided offline full-block comparator context and did not replace block-level supervised models.
Temporally resolved diagnostics	Score-history analyses for prospective temporally resolved evaluation	No validated prospective temporally resolved monitoring protocol was selected. 25 s horizons often behaved as end-of-block decisions.	Contextualised prospective temporally resolved monitoring as future work.

Note: Metrics are displayed to three decimal places. Comparisons contextualise protocol selection, representation choices, and non-selected routes. They do not introduce additional primary evaluation claims. ECG = electrocardiography. RP = recurrence plot. LOTO = leave-one-round-out. Low/high = Control plus N0 versus N1 plus N2. Three-level endpoint A = Control plus N0, with N1 and N2 separate. Three-level endpoint B = Control and N0 separate, with N1 plus N2.

Supplementary Table S2. Uncertainty estimates and exploratory paired comparisons across workload endpoints in the four-fold LOTO evaluation.

Panel A. Block-level model performance with participant-clustered 95% confidence intervals.

Endpoint	Model	Accuracy (95% CI)	Macro-F1 (95% CI)	Balanced acc. (95% CI)
Four-class	Raw ECG	0.772 (0.713–0.835)	0.771 (0.711–0.834)	0.773 (0.716–0.836)
Four-class	RP-derived	0.649 (0.593–0.702)	0.648 (0.588–0.702)	0.650 (0.594–0.706)
Four-class	Hybrid	0.754 (0.690–0.813)	0.753 (0.690–0.813)	0.754 (0.691–0.812)
Four-class	HRV-RF	0.358 (0.299–0.418)	0.358 (0.299–0.416)	0.359 (0.299–0.419)
Primary low/high	Raw ECG	0.866 (0.810–0.919)	0.865 (0.809–0.919)	0.865 (0.809–0.918)
Primary low/high	RP-derived	0.765 (0.710–0.824)	0.765 (0.709–0.825)	0.765 (0.710–0.824)
Primary low/high	Hybrid	0.847 (0.790–0.902)	0.847 (0.790–0.901)	0.847 (0.788–0.901)
Primary low/high	HRV-RF	0.649 (0.588–0.710)	0.648 (0.585–0.710)	0.649 (0.588–0.710)
Three-level A	Raw ECG	0.720 (0.662–0.784)	0.662 (0.568–0.751)	0.624 (0.550–0.711)
Three-level A	RP-derived	0.687 (0.621–0.750)	0.654 (0.579–0.726)	0.653 (0.578–0.725)
Three-level A	Hybrid	0.754 (0.698–0.813)	0.707 (0.628–0.782)	0.678 (0.608–0.755)
Three-level A	HRV-RF	0.541 (0.485–0.593)	0.450 (0.379–0.516)	0.449 (0.390–0.510)
Three-level B	Raw ECG	0.784 (0.728–0.840)	0.749 (0.671–0.819)	0.718 (0.649–0.789)
Three-level B	RP-derived	0.728 (0.665–0.788)	0.711 (0.644–0.774)	0.704 (0.640–0.768)
Three-level B	Hybrid	0.799 (0.754–0.846)	0.775 (0.717–0.832)	0.750 (0.694–0.809)
Three-level B	HRV-RF	0.474 (0.424–0.518)	0.377 (0.321–0.429)	0.387 (0.343–0.432)

Panel B. Pairwise model-difference confidence intervals.

Four-class

Metric	Comparison	Diff.	95% CI	Relation to zero
Accuracy	Raw ECG vs Hybrid	0.019	-0.022 to 0.063	Includes zero
Accuracy	Raw ECG vs RP-derived	0.123	0.072 to 0.178	Excludes zero
Accuracy	Hybrid vs RP-derived	0.104	0.072 to 0.134	Excludes zero
Accuracy	Raw ECG vs HRV-RF	0.414	0.345 to 0.492	Excludes zero
Accuracy	RP-derived vs HRV-RF	0.291	0.199 to 0.382	Excludes zero
Accuracy	Hybrid vs HRV-RF	0.396	0.306 to 0.481	Excludes zero
Macro-F1	Raw ECG vs Hybrid	0.019	-0.022 to 0.065	Includes zero
Macro-F1	Raw ECG vs RP-derived	0.123	0.070 to 0.181	Excludes zero
Macro-F1	Hybrid vs RP-derived	0.105	0.074 to 0.135	Excludes zero
Macro-F1	Raw ECG vs HRV-RF	0.413	0.342 to 0.497	Excludes zero
Macro-F1	RP-derived vs HRV-RF	0.290	0.199 to 0.383	Excludes zero
Macro-F1	Hybrid vs HRV-RF	0.395	0.307 to 0.485	Excludes zero
Balanced acc.	Raw ECG vs Hybrid	0.019	-0.022 to 0.063	Includes zero
Balanced acc.	Raw ECG vs RP-derived	0.123	0.072 to 0.178	Excludes zero
Balanced acc.	Hybrid vs RP-derived	0.104	0.072 to 0.134	Excludes zero
Balanced acc.	Raw ECG vs HRV-RF	0.414	0.343 to 0.489	Excludes zero
Balanced acc.	RP-derived vs HRV-RF	0.291	0.200 to 0.378	Excludes zero
Balanced acc.	Hybrid vs HRV-RF	0.395	0.306 to 0.480	Excludes zero

Primary low/high

Metric	Comparison	Diff.	95% CI	Relation to zero
Accuracy	Raw ECG vs Hybrid	0.019	-0.022 to 0.056	Includes zero
Accuracy	Raw ECG vs RP-derived	0.101	0.052 to 0.152	Excludes zero
Accuracy	Hybrid vs RP-derived	0.082	0.051 to 0.115	Excludes zero
Accuracy	Raw ECG vs HRV-RF	0.216	0.129 to 0.307	Excludes zero
Accuracy	RP-derived vs HRV-RF	0.116	0.022 to 0.215	Excludes zero
Accuracy	Hybrid vs HRV-RF	0.198	0.114 to 0.287	Excludes zero
Macro-F1	Raw ECG vs Hybrid	0.018	-0.022 to 0.056	Includes zero
Macro-F1	Raw ECG vs RP-derived	0.100	0.052 to 0.151	Excludes zero
Macro-F1	Hybrid vs RP-derived	0.082	0.051 to 0.116	Excludes zero
Macro-F1	Raw ECG vs HRV-RF	0.217	0.130 to 0.309	Excludes zero
Macro-F1	RP-derived vs HRV-RF	0.117	0.024 to 0.215	Excludes zero
Macro-F1	Hybrid vs HRV-RF	0.199	0.117 to 0.289	Excludes zero
Balanced acc.	Raw ECG vs Hybrid	0.018	-0.023 to 0.055	Includes zero
Balanced acc.	Raw ECG vs RP-derived	0.100	0.051 to 0.151	Excludes zero
Balanced acc.	Hybrid vs RP-derived	0.082	0.051 to 0.116	Excludes zero
Balanced acc.	Raw ECG vs HRV-RF	0.217	0.130 to 0.306	Excludes zero
Balanced acc.	RP-derived vs HRV-RF	0.117	0.024 to 0.214	Excludes zero
Balanced acc.	Hybrid vs HRV-RF	0.199	0.114 to 0.288	Excludes zero

Three-level A

Metric	Comparison	Diff.	95% CI	Relation to zero
Accuracy	Raw ECG vs Hybrid	-0.034	-0.070 to 0.000	Includes zero
Accuracy	Raw ECG vs RP-derived	0.034	-0.011 to 0.077	Includes zero
Accuracy	Hybrid vs RP-derived	0.067	0.026 to 0.103	Excludes zero
Accuracy	Raw ECG vs HRV-RF	0.179	0.088 to 0.277	Excludes zero
Accuracy	RP-derived vs HRV-RF	0.146	0.051 to 0.246	Excludes zero
Accuracy	Hybrid vs HRV-RF	0.213	0.121 to 0.306	Excludes zero
Macro-F1	Raw ECG vs Hybrid	-0.045	-0.105 to 0.005	Includes zero
Macro-F1	Raw ECG vs RP-derived	0.008	-0.054 to 0.061	Includes zero
Macro-F1	Hybrid vs RP-derived	0.053	0.011 to 0.090	Excludes zero
Macro-F1	Raw ECG vs HRV-RF	0.212	0.088 to 0.335	Excludes zero
Macro-F1	RP-derived vs HRV-RF	0.205	0.099 to 0.318	Excludes zero
Macro-F1	Hybrid vs HRV-RF	0.257	0.144 to 0.378	Excludes zero
Balanced acc.	Raw ECG vs Hybrid	-0.054	-0.100 to -0.011	Excludes zero
Balanced acc.	Raw ECG vs RP-derived	-0.028	-0.075 to 0.017	Includes zero
Balanced acc.	Hybrid vs RP-derived	0.025	-0.012 to 0.060	Includes zero
Balanced acc.	Raw ECG vs HRV-RF	0.175	0.069 to 0.287	Excludes zero
Balanced acc.	RP-derived vs HRV-RF	0.203	0.103 to 0.307	Excludes zero
Balanced acc.	Hybrid vs HRV-RF	0.229	0.123 to 0.339	Excludes zero

Three-level B

Metric	Comparison	Diff.	95% CI	Relation to zero
Accuracy	Raw ECG vs Hybrid	-0.015	-0.061 to 0.030	Includes zero
Accuracy	Raw ECG vs RP-derived	0.056	-0.015 to 0.129	Includes zero
Accuracy	Hybrid vs RP-derived	0.071	0.030 to 0.108	Excludes zero
Accuracy	Raw ECG vs HRV-RF	0.310	0.257 to 0.362	Excludes zero
Accuracy	RP-derived vs HRV-RF	0.254	0.172 to 0.335	Excludes zero
Accuracy	Hybrid vs HRV-RF	0.325	0.261 to 0.390	Excludes zero
Macro-F1	Raw ECG vs Hybrid	-0.026	-0.085 to 0.031	Includes zero
Macro-F1	Raw ECG vs RP-derived	0.038	-0.048 to 0.121	Includes zero
Macro-F1	Hybrid vs RP-derived	0.064	0.017 to 0.103	Excludes zero
Macro-F1	Raw ECG vs HRV-RF	0.372	0.289 to 0.455	Excludes zero
Macro-F1	RP-derived vs HRV-RF	0.334	0.230 to 0.431	Excludes zero
Macro-F1	Hybrid vs HRV-RF	0.398	0.305 to 0.491	Excludes zero
Balanced acc.	Raw ECG vs Hybrid	-0.031	-0.085 to 0.022	Includes zero
Balanced acc.	Raw ECG vs RP-derived	0.014	-0.064 to 0.096	Includes zero
Balanced acc.	Hybrid vs RP-derived	0.046	0.004 to 0.083	Excludes zero
Balanced acc.	Raw ECG vs HRV-RF	0.331	0.257 to 0.405	Excludes zero
Balanced acc.	RP-derived vs HRV-RF	0.317	0.223 to 0.401	Excludes zero
Balanced acc.	Hybrid vs HRV-RF	0.362	0.279 to 0.443	Excludes zero

Panel C. Paired randomisation-test grid.

Four-class

Metric	Comparison	Diff.	p	Holm p	Global p	Flag
Accuracy	Raw ECG vs Hybrid	0.019	0.527	0.527	1.000	numerical only
Accuracy	Raw ECG vs RP-derived	0.123	0.000488	0.000977	0.022	exploratory Holm
Accuracy	Hybrid vs RP-derived	0.104	0.000137	0.000412	0.007	exploratory Holm
Accuracy	Raw ECG vs HRV-RF	0.414	0.0000153	0.0000916	0.001	exploratory Holm
Accuracy	RP-derived vs HRV-RF	0.291	0.0000916	0.000366	0.005	exploratory Holm
Accuracy	Hybrid vs HRV-RF	0.396	0.0000305	0.000153	0.002	exploratory Holm
Macro-F1	Raw ECG vs Hybrid	0.019	0.468	0.468	1.000	numerical only
Macro-F1	Raw ECG vs RP-derived	0.123	0.000427	0.000854	0.020	exploratory Holm
Macro-F1	Hybrid vs RP-derived	0.105	0.0000763	0.000305	0.005	exploratory Holm
Macro-F1	Raw ECG vs HRV-RF	0.413	0.0000153	0.0000916	0.001	exploratory Holm
Macro-F1	RP-derived vs HRV-RF	0.290	0.0000763	0.000305	0.005	exploratory Holm
Macro-F1	Hybrid vs HRV-RF	0.395	0.0000305	0.000153	0.002	exploratory Holm
Bal. acc.	Raw ECG vs Hybrid	0.019	0.479	0.479	1.000	numerical only
Bal. acc.	Raw ECG vs RP-derived	0.123	0.000320	0.000641	0.016	exploratory Holm
Bal. acc.	Hybrid vs RP-derived	0.104	0.000137	0.000412	0.007	exploratory Holm
Bal. acc.	Raw ECG vs HRV-RF	0.414	0.0000153	0.0000916	0.001	exploratory Holm
Bal. acc.	RP-derived vs HRV-RF	0.291	0.0000916	0.000366	0.005	exploratory Holm
Bal. acc.	Hybrid vs HRV-RF	0.395	0.0000305	0.000153	0.002	exploratory Holm

Primary low/high

Metric	Comparison	Diff.	p	Holm p	Global p	Flag
Accuracy	Raw ECG vs Hybrid	0.019	0.486	0.486	1.000	numerical only
Accuracy	Raw ECG vs RP-derived	0.101	0.002	0.006	0.064	exploratory Holm
Accuracy	Hybrid vs RP-derived	0.082	0.000488	0.003	0.022	exploratory Holm
Accuracy	Raw ECG vs HRV-RF	0.216	0.000488	0.003	0.022	exploratory Holm
Accuracy	RP-derived vs HRV-RF	0.116	0.042	0.084	0.823	nominal only
Accuracy	Hybrid vs HRV-RF	0.198	0.000732	0.003	0.028	exploratory Holm
Macro-F1	Raw ECG vs Hybrid	0.018	0.485	0.485	1.000	numerical only
Macro-F1	Raw ECG vs RP-derived	0.100	0.002	0.005	0.061	planned support
Macro-F1	Hybrid vs RP-derived	0.082	0.000488	0.002	0.022	planned support
Macro-F1	Raw ECG vs HRV-RF	0.217	0.000366	0.002	0.018	planned support
Macro-F1	RP-derived vs HRV-RF	0.117	0.030	0.060	0.694	nominal only
Macro-F1	Hybrid vs HRV-RF	0.199	0.000366	0.002	0.018	planned support
Bal. acc.	Raw ECG vs Hybrid	0.018	0.485	0.485	1.000	numerical only
Bal. acc.	Raw ECG vs RP-derived	0.100	0.002	0.005	0.061	planned support
Bal. acc.	Hybrid vs RP-derived	0.082	0.000488	0.002	0.022	planned support
Bal. acc.	Raw ECG vs HRV-RF	0.217	0.000366	0.002	0.018	planned support
Bal. acc.	RP-derived vs HRV-RF	0.117	0.030	0.060	0.694	nominal only
Bal. acc.	Hybrid vs HRV-RF	0.199	0.000366	0.002	0.018	planned support

Three-level A

Metric	Comparison	Diff.	p	Holm p	Global p	Flag
Accuracy	Raw ECG vs Hybrid	-0.034	0.133	0.266	1.000	numerical only
Accuracy	Raw ECG vs RP-derived	0.034	0.218	0.266	1.000	numerical only
Accuracy	Hybrid vs RP-derived	0.067	0.010	0.042	0.283	exploratory Holm
Accuracy	Raw ECG vs HRV-RF	0.179	0.002	0.010	0.064	exploratory Holm
Accuracy	RP-derived vs HRV-RF	0.146	0.015	0.045	0.392	exploratory Holm
Accuracy	Hybrid vs HRV-RF	0.213	0.000488	0.003	0.022	exploratory Holm
Macro-F1	Raw ECG vs Hybrid	-0.045	0.135	0.270	1.000	numerical only
Macro-F1	Raw ECG vs RP-derived	0.008	0.791	0.791	1.000	numerical only
Macro-F1	Hybrid vs RP-derived	0.053	0.023	0.068	0.545	nominal only
Macro-F1	Raw ECG vs HRV-RF	0.212	0.004	0.017	0.126	exploratory Holm
Macro-F1	RP-derived vs HRV-RF	0.205	0.003	0.013	0.081	exploratory Holm
Macro-F1	Hybrid vs HRV-RF	0.257	0.000610	0.004	0.024	exploratory Holm
Bal. acc.	Raw ECG vs Hybrid	-0.054	0.041	0.122	0.823	nominal only
Bal. acc.	Raw ECG vs RP-derived	-0.028	0.282	0.420	1.000	numerical only
Bal. acc.	Hybrid vs RP-derived	0.025	0.210	0.420	1.000	numerical only
Bal. acc.	Raw ECG vs HRV-RF	0.175	0.007	0.026	0.189	exploratory Holm
Bal. acc.	RP-derived vs HRV-RF	0.203	0.001	0.007	0.049	exploratory Holm
Bal. acc.	Hybrid vs HRV-RF	0.229	0.001	0.006	0.039	exploratory Holm

Three-level B

**Metric**	**Comparison**	**Diff.**	**p**	**Holm p**	**Global p**	**Flag**
Accuracy	Raw ECG vs Hybrid	-0.015	0.646	0.646	1.000	numerical only
Accuracy	Raw ECG vs RP-derived	0.056	0.190	0.380	1.000	numerical only
Accuracy	Hybrid vs RP-derived	0.071	0.008	0.024	0.226	exploratory Holm
Accuracy	Raw ECG vs HRV-RF	0.310	0.0000153	0.0000916	0.001	exploratory Holm
Accuracy	RP-derived vs HRV-RF	0.254	0.000153	0.000610	0.008	exploratory Holm
Accuracy	Hybrid vs HRV-RF	0.325	0.0000153	0.0000916	0.001	exploratory Holm
Macro-F1	Raw ECG vs Hybrid	-0.026	0.393	0.786	1.000	numerical only
Macro-F1	Raw ECG vs RP-derived	0.038	0.399	0.786	1.000	numerical only
Macro-F1	Hybrid vs RP-derived	0.064	0.015	0.046	0.392	exploratory Holm
Macro-F1	Raw ECG vs HRV-RF	0.372	0.0000153	0.0000916	0.001	exploratory Holm
Macro-F1	RP-derived vs HRV-RF	0.334	0.0000763	0.000305	0.005	exploratory Holm
Macro-F1	Hybrid vs HRV-RF	0.398	0.0000153	0.0000916	0.001	exploratory Holm
Bal. acc.	Raw ECG vs Hybrid	-0.031	0.307	0.614	1.000	numerical only
Bal. acc.	Raw ECG vs RP-derived	0.014	0.731	0.731	1.000	numerical only
Bal. acc.	Hybrid vs RP-derived	0.046	0.039	0.118	0.823	nominal only
Bal. acc.	Raw ECG vs HRV-RF	0.331	0.0000153	0.0000916	0.001	exploratory Holm
Bal. acc.	RP-derived vs HRV-RF	0.317	0.0000916	0.000366	0.005	exploratory Holm
Bal. acc.	Hybrid vs HRV-RF	0.362	0.0000153	0.0000916	0.001	exploratory Holm

Note: Point estimates are from pooled held-out block-level predictions from the four-fold LOTO evaluation. CIs and paired tests preserve participant clustering. Panel A reports participant-clustered percentile 95% CIs. Panel B reports paired participant-clustered model-difference CIs, with differences computed as first-listed model minus second-listed model. Panel C reports exact participant-clustered paired randomisation tests. Only primary low/high macro-F1 and balanced-accuracy comparisons are planned main-text tests; all remaining rows are exploratory. p denotes the unadjusted paired-test p-value. Holm p denotes correction within each endpoint-metric family. Global p denotes the exploratory global all-endpoint family when available. Global p is reported for exploratory context only and is not used to determine planned support for the primary low/high comparisons. Planned support denotes primary low/high planned main-text support. Exploratory Holm denotes support within the displayed exploratory endpoint-metric family only. Nominal only denotes unadjusted evidence that did not remain supported after Holm correction. Numerical only denotes no paired-test support. Raw ECG, RP-derived, and hybrid are representation branches. CI = confidence interval. HRV-RF = heart-rate/time-domain heart-rate-variability Random Forest. LOTO = leave-one-round-out. acc. = accuracy. Bal. acc. = balanced accuracy. The full all-endpoint p-value grid is exploratory and is not used to claim universal branch superiority.
